# Concurrent Generation of Tight and Loose Ion Pairs upon Charge-Transfer Excitation of Electron Donor–Acceptor Complexes in Solution

**DOI:** 10.1021/acs.jpclett.5c03709

**Published:** 2026-01-26

**Authors:** Guan-Yu Chen, Yi-Kai Liao, Pin-Hsun Chen, Yu-Cheng Hsu, Pei-Chen Chiang, Yu-Chen Hsu, Bo-Yu Chang, Guan-Sho Chen, Yi-Fan Wen, Yu-Fang Yeh, Wen-Teng Hsu, Chih-Chang Hung, Chih-Chung Chiu, Po-Yuan Cheng

**Affiliations:** Department of Chemistry, 34881 National Tsing Hua University, Hsinchu 300044, Taiwan, Republic of China

## Abstract

Ultrafast time-resolved fluorescence (TRFL) and visible transient absorption (TA) measurements were performed to investigate excited-state dynamics of several electron donor–acceptor complexes (DACs) following charge-transfer (CT) excitation in solution. For all DACs studied, including benzene–tetracyanoethylene (BZ–TCNE), toluene–TCNE, fluorobenzene–TCNE, and (BZ)_2_–TCNE, the CT-state lifetimes obtained from TRFL are consistently shorter than those from TA by factors of ∼2–5. This disparity, together with fluorescence lifetimes of ∼5–30 ps in dichloromethane, cannot be reconciled with the conventional assumption that CT excitation initially yields only emissive tight ion pairs (TIPs). The results instead support a revised concurrent mechanism in which CT excitation generates a locally hot and structurally diverse ion-pair ensemble that bifurcates into parallel relaxation pathways, leading to fluorescent TIPs and nonfluorescent loose ion pairs. The two species undergo independent charge recombination at different rates, yielding the distinct lifetimes observed in the TRFL and TA measurements.

## Graphical abstract

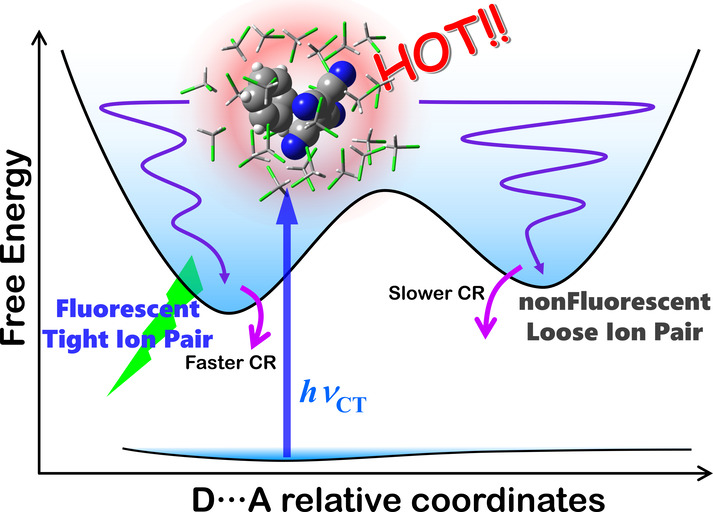

Electron donor–acceptor complexes (DACs) serve as valuable models for probing intermolecular energy and electron transfer in various natural and artificial systems.^1–7^ Formation of DACs in solution is often manifested by the emergence of a low-energy charge-transfer (CT) absorption band that is absent in either the donor (D) or the acceptor (A).^8–11^ Optical excitation of a DAC within its CT band results in an excited state with a high degree of charge separation that is well described as a radical ion pair (IP).^11–14^ Because CT excitations require substantial overlap between the frontier molecular orbitals (MOs) of D and A to attain appreciable oscillator strength, it is generally assumed that only DAC configurations with strong electronic coupling are optically excited.^15–20^ Consequently, CT excitation of DACs has been widely used as a direct route to generate closely contacted IPs for probing their subsequent dynamics.^21–38^

Among various DAC systems, the benzene–tetracyanoethylene (BZ–TCNE) complex represents a prototypical model. We have investigated its CT-state relaxation dynamics following CT excitation using broadband ultrafast time-resolved fluorescence (TRFL) spectroscopy in solvents of different polarity.^37,38^ Analysis of various time-dependent spectral properties revealed rapid solvation and vibrational relaxation, followed by slower charge recombination (CR).^37,38^ Recently, Rumble and Vauthey reported ultrafast visible and IR transient absorption (TA) measurements on BZ–TCNE in dichloromethane (CH_2_Cl_2_).^35^ They observed a CT-state lifetime of ∼55–60 ps, nearly twice our previous TRFL value of ∼29 ps in the same solvent.^37^ Together, these results indicate a disparity between the CT-state lifetimes measured by TRFL and TA.

To investigate the origin of this discrepancy, we performed combined ultrafast TRFL and visible TA measurements following CT excitation of five DAC systems. The first three are binary BZ–TCNE, toluene (TL)–TCNE, and fluorobenzene (FB)–TCNE complexes in medium-polarity solvent CH_2_Cl_2_, while the other two compare binary and ternary BZ/TCNE complexes in the nonpolar solvent tetrachloromethane (CCl_4_). The spectroscopic setups for the TRFL and TA measurements have been described elsewhere,^37–40^ and additional experimental details are provided in the Supporting Information (SI). The steady-state CT absorption spectra of the five DAC systems are shown in Figure S1. By exciting these DAC systems near the maxima of their CT absorption bands and recording their TRFL and TA spectra, we found that the CT-state lifetimes obtained from TRFL are consistently ∼2–5 times shorter than those determined by TA. This Letter focuses on elucidating the origin of this disparity.

Figure 1a–d show the TRFL and TA spectra and transients of BZ–TCNE excited within its CT band in CH_2_Cl_2_. The TRFL spectra, which are the same data reported previously,^37^ undergo rapid spectral evolution due to solvation and vibrational/structural relaxation within the first few picoseconds, followed by a much slower intensity decay. These spectra were reanalyzed by global fitting using the Glotaran program,^41^ and singular value decomposition indicated that at least four exponential components are required to describe the data. The decay-associated spectra (DAS) resolved from the fit are shown in Figure S5, and the extracted time constants are summarized in Table 1. The two subpicosecond components are assigned to solvation, while τ_3F_ and τ_F_ agree well with the values obtained previously from total fluorescence intensity analyses.^37^ τ_3F_ is attributed to vibrational and structural relaxation, and τ_F_ (29 ps) corresponds to the CT-state fluorescence lifetime.

**Table 1:** Time Constants^a^ Obtained from Global Fits of TRFL and TA Spectra for Five DAC Systems

	time constants (ps) from TRFL				time constants (ps) from TA					
DAC system^b^	τ_1F_	τ_2F_	τ_3F_	**τ**_**F**_	τ_1A_	τ_2A_	τ_3A_	τ_4A_	**τ**_**A**_	τ_GSB_
BZ-TCNE in CH_2_Cl_2_	0.2	0.6	3.1	**29**	<0.2	0.5	4.1	29^c^	**62**	
TL-TCNE in CH_2_Cl_2_	<0.2	0.7		**4.5**	<0.2	0.6	4.2		**10.5**	72
FB-TCNE in CH_2_Cl_2_	0.2	0.6		**5.3**	<0.2		4.4		**27**	
BZ-TCNE in CCl_4_	0.4	2.4	17	**153**	0.3		9.9	153^c^	**403**	
BZ/TCNE in 10% BZ/CCl_4_ ^d^	0.2	1.5	10	**59**	<0.2		3.9	27	**208**	

a Relative uncertainties are 10–30% for subpicosecond components and 2–10% for longer ones, depending on the time scale and signal-to-noise ratio.

b Concentrations of aromatic donors and TCNE are given in Table S1.

c This time constant is fixed at corresponding τ_F_. Values without this restriction are τ_4A_ = 12 ps and τ_A_ = 59 ps for BZ–TCNE in CH_2_Cl_2_; τ_4A_ = 75 ps and τ_A_ = 392 ps for BZ–TCNE in CCl_4_.

d This solution contains binary and ternary (D_2_A) complexes.

**Figure 1:**
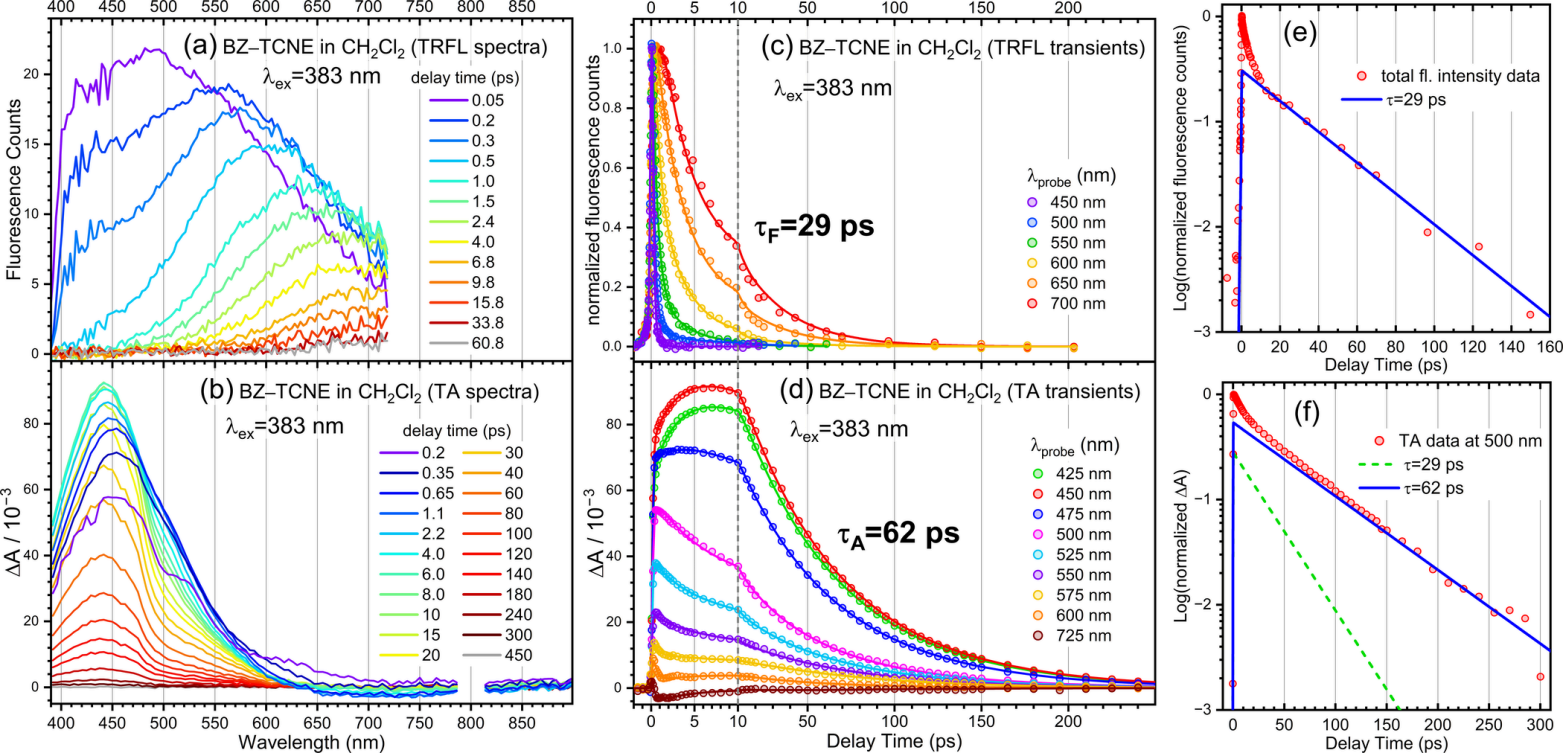
TRFL and TA data of BZ–TCNE in CH_2_Cl_2_. (a) TRFL and (b) TA wavelength spectra at selected delay times. (c) TRFL and (d) TA transients at selected wavelengths; solid lines are results of global fits. (e) Semilog plot of the time-dependent total fluorescence intensity derived from the TRFL spectra. The solid line is the 29 ps component obtained from a multiexponential fit. (f) Semilog plot of the TA transient at 500 nm. The green and blue lines are the 29 and 62 ps components, respectively, obtained from a multiexponential fit.

The TA spectra (Figure 1b) exhibit a dominant excited-state absorption (ESA) band spanning 350–600 nm, with a maximum near 450 nm. This band exhibits a rapid rise following CT excitation and is assigned to the ESA of the CT state, which is best described as an ion pair (IP). This assignment is supported by the close resemblance of the 450 nm ESA band to the visible absorption of the TCNE anion.^42,43^ The counter BZ cation also absorbs in this region, although with a much smaller extinction coefficient.^44,45^ The 450 nm ESA band exhibits a slight blue shift during the first few picoseconds, followed by slower spectral narrowing. These early spectral evolutions are attributed to solvation and vibrational/structural relaxation, analogous to those observed in the TRFL spectra.

Comparison of the TRFL and TA data reveals distinct decay time scales. At ∼60 ps, the fluorescence signal has decayed to a negligible level, whereas the 450 nm ESA band still retains roughly half of its maximum intensity. In the 650–850 nm region of the TA spectra, a very weak stimulated emission (SE) band is present. Its blue side is obscured by the ESA band, but the red side clearly shows a faster decay than the ESA signal (Figure S2).

Global analysis of the TA spectra required five components to reproduce the data (Figure S5 and Table 1). The first three components (τ_1A_–τ_3A_) are similar to those resolved in TRFL spectra. The negative DAS associated with τ_1A_ (Figure S5) indicates that the TCNE^–^ absorption does not rise instantaneously, consistent with an earlier observation.^26^ One component (τ_4A_) was fixed at 29 ps to account for the fluorescent IP population. The need to include this component is justified by the semilog plots shown in Figure 1e and 1f, which reveal that, beyond ∼10 ps, the TA signal at 500 nm exhibits a biexponential decay, whereas the total fluorescence intensity decays single-exponentially. The slowest component (τ_A_ = 62 ps) resolved here is slightly longer than the ∼55–60 ps lifetime reported previously by Rumble and Vauthey.^35^

TRFL and TA spectra of the other DAC systems are shown in Figures 2 and 3. All TRFL and TA data were analyzed by global fitting. The resolved DASs are listed in Figure S5, and the extracted time constants are summarized in Table 1. In all cases, the CT-state lifetimes derived from TRFL and TA measurements are denoted as τ_F_ and τ_A_, respectively. In the following, only the key features of each system are described.

**Figure 2:**
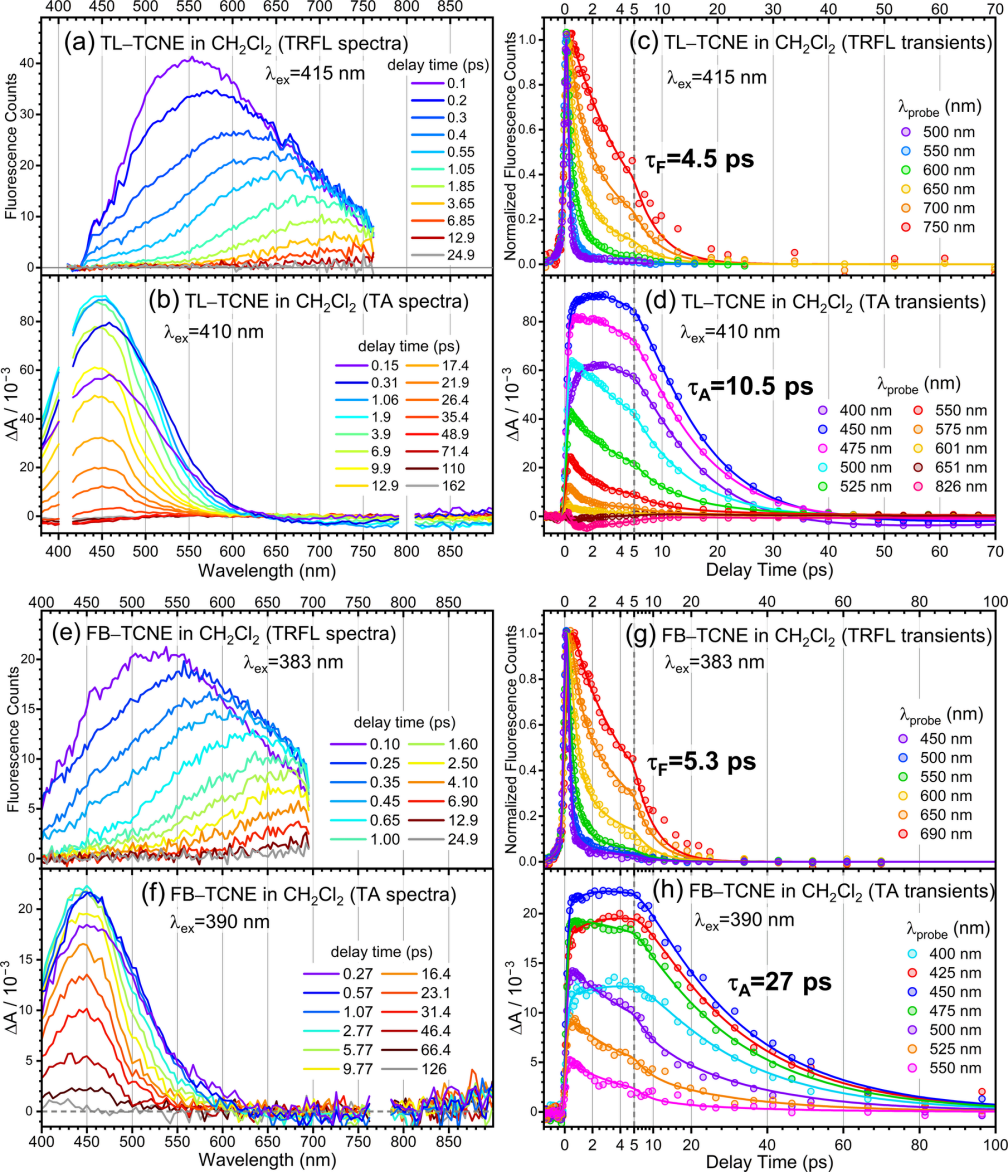
(a) TRFL and (b) TA wavelength spectra of TL–TCNE in CH_2_Cl_2_ at selected delay times. (c) TRFL and (d) TA transients of TL–TCNE in CH_2_Cl_2_ at selected wavelengths; solid lines are results of global fits. (e) TRFL and (f) TA wavelength spectra of FB–TCNE in CH_2_Cl_2_ at selected delay times. (g) TRFL and (h) TA transients of FB–TCNE in CH_2_Cl_2_ at selected wavelengths; solid lines are results of global fits.

**Figure 3:**
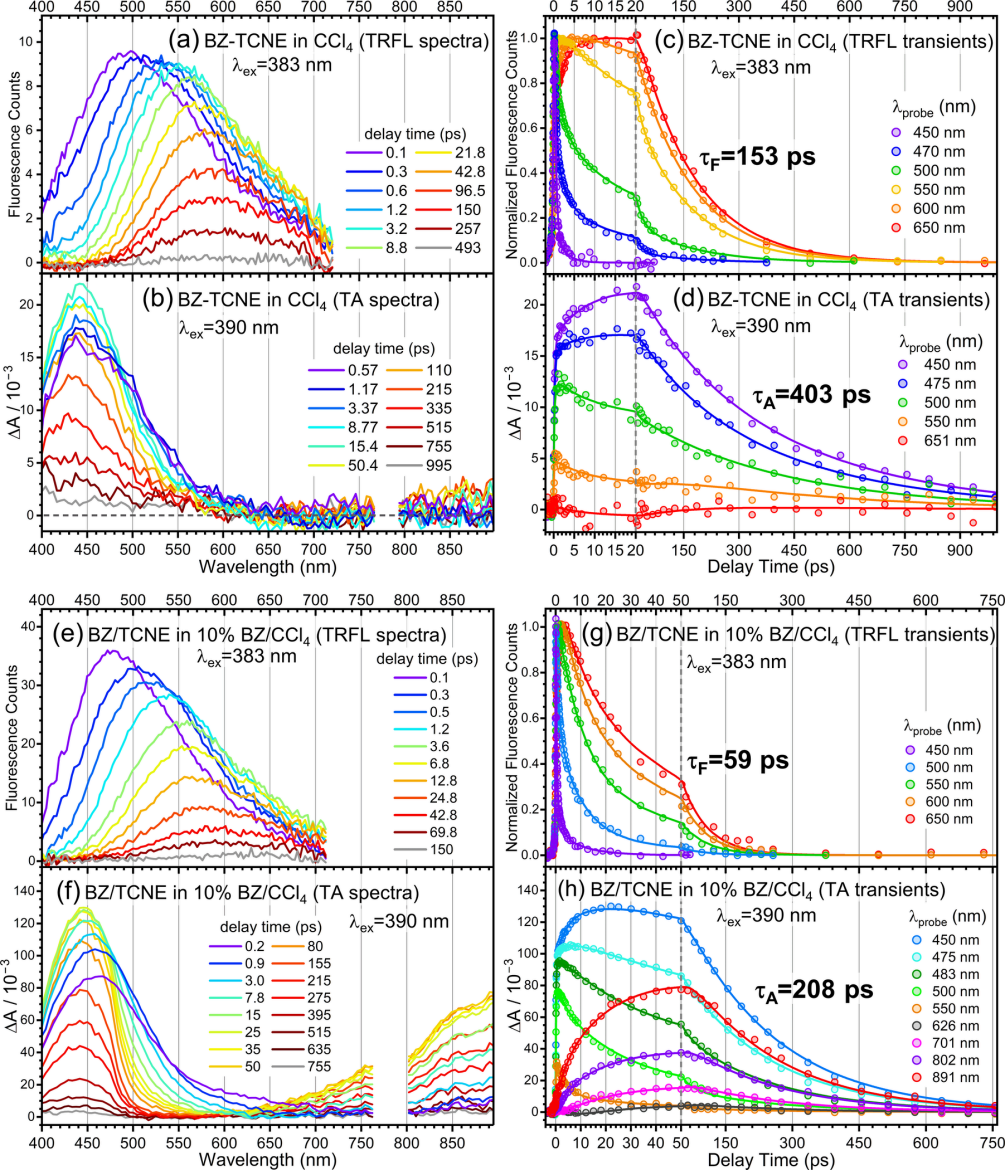
(a) TRFL and (b) TA wavelength spectra of BZ–TCNE in CCl_4_ at selected delay times. (c) TRFL and (d) TA transients of BZ–TCNE in CCl_4_ at selected wavelengths; solid lines are results of global fits. (e) TRFL and (f) TA wavelength spectra of BZ/TCNE complexes in 10% BZ/CCl_4_ at selected delay times. (g) TRFL and (h) TA transients of BZ/TCNE complexes in 10% BZ/CCl_4_ at selected wavelengths; solid lines are results of global fits.

For TL–TCNE in CH_2_Cl_2_, the reduced driving force for CR is expected to result in a faster CR rate. Global analysis of the TRFL data (Figure 2a and 2c) yields τ_F_ = 4.5 ps, which can be ascribed to vibrational nonequilibrium CR,^30,46^ as this time scale is comparable to vibrational relaxation. The TA data shown in Figure 2b and 2d again exhibit a slower decay. Global analysis indicates that at least five components are required, including a 4.2 ps component close to τ_F_ and a 10.5 ps decay (τ_A_) associated with the 450 nm ESA band. The value of τ_A_ resolved here is consistent with earlier studies.^47,48^ The slowest component (∼72 ps) corresponds to recovery of the ground-state bleach (GSB) band in a spectral region matching the ground-state absorption (Figure S3). A weak SE band between 650 and 890 nm, consistent with the TRFL data, is also observed (Figure S3).

Figure 2e–h show the TRFL and TA data of FB–TCNE in CH_2_Cl_2_. As in the other systems, the fluorescence decays much faster than the TA signal. Global analysis yields τ_F_ = 5.3 ps and τ_A_ = 27 ps. The former is also attributed to vibrational nonequilibrium CR.^30,46^ Evidently, the lifetime measured by TA is substantially longer than that obtained from TRFL.

For BZ–TCNE in CCl_4_ (Figure 3a–d), the weaker solvation in nonpolar solvent increases the energy gap and reduces the CR rate, resulting in a much longer fluorescence lifetime of τ_F_ = 153 ps.^37,38^ The TA spectra are likewise dominated by the 450 nm ESA band. Global analysis gives τ_A_ = 403 ps, indicating that the BZ–TCNE CT-state lifetime observed by TA in nonpolar solvent is also substantially longer than that measured by TRFL. The need to include a component (τ_4A_) to account for fluorescent IPs is justified by the semilog plots shown in Figure S4.

Figure 3e and 3g present the TRFL data for BZ/TCNE^49^ complexes in a 10% BZ/CCl_4_ cosolvent.^50^ As concluded previously,^38^ although 2:1 ternary complexes are present in this solution, the initial CT excitation arises primarily from 1:1 binary complexes, whereas the fluorescence is dominated by emission from the asymmetric 2:1 CT state, (BZ)_2_^+^–TCNE^–^, produced by secondary formation involving diffusion. Charge resonance in the BZ dimer cation ((BZ)_2_^+^) reduces the CR exothermicity and increases the CR rate, leading to a shorter fluorescence lifetime relative to that in CCl_4_.^38^ The TRFL spectra were reanalyzed by global fitting to characterize the temporal evolution of the TRFL spectra, without addressing the individual dynamics of 1:1 and 2:1 CT states described previously.^38^ Nevertheless, τ_F_ = 59 ps is comparable to the ∼70 ps lifetime derived for the 2:1 CT state from a more sophisticated kinetic model.^38^

TA spectra of BZ/TCNE^49^ complexes in a 10% BZ/CCl_4_ cosolvent^50^ are shown in Figure 3f. At early delay times (<1 ps), the spectra are dominated by the 450 nm ESA band. Beyond ∼1 ps, a new long-wavelength ESA band (>650 nm) grows in steadily, reaching a maximum at ∼50 ps before decaying slowly in parallel with the 450 nm band (Figure 3h). This long-wavelength ESA band is consistent with the characteristic charge-resonance absorption of (BZ)_2_^+^, which peaks near 900 nm.^44,51^ Global analysis of the TA spectra reveals a 27 ps process likely associated with secondary formation and a 208 ps slow decay (τ_A_) of the (BZ)_2_^+^ and TCNE^–^ absorption bands. The concurrent decay of these two bands unequivocally indicates that this decay arises from the CR of asymmetric 2:1 CT complexes. Again, τ_A_ is much longer than τ_F_ even for ternary IPs.

In this work, we focus primarily on the pronounced difference between the CT-state lifetimes measured by TRFL (τ_F_) and TA (τ_A_). As summarized in Table 1, τ_F_ is consistently shorter than τ_A_ by factors of ∼2–5 in all systems studied. The first three binary DACs in CH_2_Cl_2_ are similar in nature, while the other two systems compare binary and ternary DACs in nonpolar solvents. Thus, the effect is robust and suggests a general phenomenon that has previously gone unnoticed.

Excitation of DACs within their CT absorption bands produces CT states that are well described as IPs (SI, section S6.2).^11–14^ TRFL monitors the emission from fluorescent IPs, whereas TA tracks the absorption of ionic species in the entire IP population. For time constants associated with solvation and vibrational relaxation, modest differences measured by the two methods are not unusual. However, because τ_F_ and τ_A_ primarily reflect excited-state population decay, the two techniques should yield similar lifetimes if the same transient species are probed. Numerous combined TRFL and TA studies, including those presented in SI section S5, have demonstrated that this agreement holds for many photochemical systems, extending even into the subpicosecond regime.^40,52–61^ Consequently, the markedly different lifetimes measured by TRFL and TA suggest the existence of a longer-lived nonfluorescent state that is invisible to TRFL but can be detected by TA. Furthermore, because TA detection in this study relies on ionic-species absorption, this nonfluorescent state must also be ionic in nature.

A triplet CT state could, in principle, account for the longer-lived nonfluorescent species. However, this would require ultrafast intersystem crossing (ISC) on time scales competitive with τ_F_, i.e., ∼5–150 ps. This assignment is highly improbable as spin–orbit coupling in CT states of DACs is vanishingly small due to their ionic character, and ISC proceeds only through weak hyperfine interactions with time scales of at least a few tens of nanoseconds.^62,63^

Thus, the markedly different lifetimes measured by TA and TRFL strongly suggest that CT excitation of DACs generates at least two distinct types of singlet IPs that differ in fluorescence efficiency and CR rate. The IPs detected by TRFL are fluorescent and exhibit faster CR, whereas those detected only by TA are nonfluorescent and undergo slower CR. These properties point to the use of electronic coupling as a descriptor for distinguishing IP types. Following the definition suggested by Vauthey and co-workers,^33,34^ the fluorescent IPs are referred to as tight IPs (TIPs), which possess strong electronic coupling and exhibit faster CR dynamics; and the longer-lived nonfluorescent IPs are referred to as loose IPs (LIPs), which are characterized by weak coupling and slower CR.

The above interpretation appears at first glance to align with the conventional sequential mechanism widely used to describe IP generation and subsequent dynamics.^11,15–25,31^ As illustrated in Figure 4a, it is conventionally assumed that, upon excitation within the CT band of DACs, initial relaxation from the emissive Franck–Condon (FC) state produces only TIPs, which subsequently convert to LIPs that are often presumed to be solvent-separated IPs (SSIPs).^11,15–25^ In favorable cases, SSIPs may undergo further solvation to produce free ions.^17^ Although the conventional mechanism may account for longer-lived TIPs in previous studies,^11,15–25^ it is incompatible with the short-lived TIPs observed here, where τ_F_ can be as short as ∼5 ps, which is much faster than the typical TIP→SSIP conversion time scale of ∼10^–9^ s for similar systems.^18,22–24^ However, because the nonfluorescent LIPs proposed here are not limited to SSIPs and may encompass a broad range of interionic orientations and separations, slow SSIP formation alone is insufficient to rule out the conventional mechanism; other intracomplex structural conversions must also be considered.

**Figure 4:**
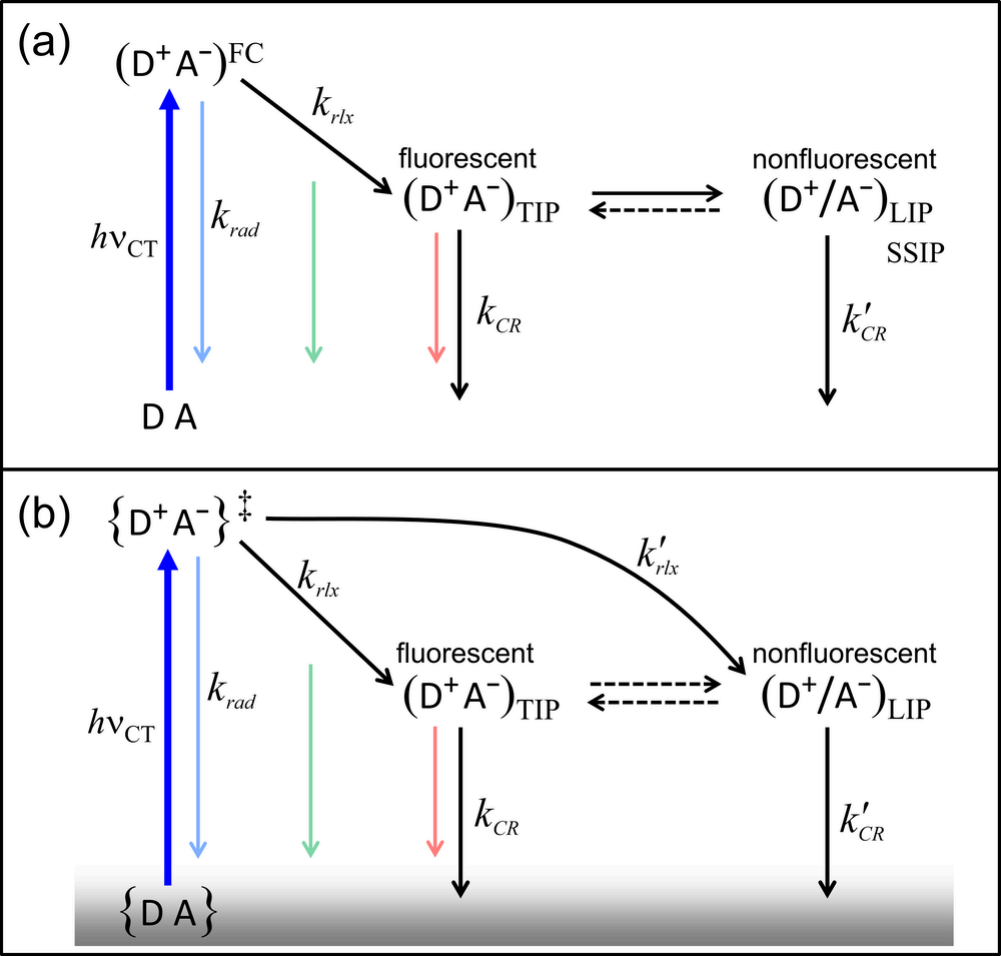
(a) Conventional sequential mechanism for IP generation and relaxation dynamics upon CT excitation of DACs. (b) Proposed concurrent mechanism to account for the observations reported in this work. {D^+^A^–^}^‡^ denotes the initial IP ensemble generated upon CT excitation of ground-state DAC ensemble {DA}. (D^+^A^–^)_TIP_ and (D^+^/A^–^)_LIP_ represent relaxed TIP and LIP, respectively. The dashed arrows indicate that equilibrium between TIPs and LIPs is established only when their interconversion rates are comparable to or faster than their decay rates.

A key implication of our data is that, regardless of the specific nature of the nonfluorescent LIPs, the conventional sequential model cannot simultaneously account for the different τ_F_ and τ_A_ observed for the three similar DAC systems in CH_2_Cl_2_. For BZ–TCNE in CH_2_Cl_2_, the fluorescence lifetime is 29 ps; therefore, if the conventional mechanism were operative, the TIP→LIP conversion would be slower, and possibly much slower, than 29 ps. In that case, the same slow conversion should also occur for TL–TCNE and FB–TCNE in the same solvent, yet such a slow process cannot compete with the observed rapid ∼5 ps decay of their TIPs. Consequently, LIP production would be minimal, and TRFL and TA should yield nearly identical lifetimes, contrary to our observations. Conversely, if the short τ_F_ (∼5 ps) of TL–TCNE and FB–TCNE TIPs were dictated by rapid TIP→LIP conversion, then an equally rapid conversion should also occur in BZ–TCNE and produce a similarly short τ_F_, which again contradicts our observations. Thus, regardless of the nature of LIPs, the conventional sequential model is incompatible with the present data. This led us to consider a revised model in which TIPs and LIPs are produced concurrently.

As illustrated in Figure 4b, we propose that CT excitation of DACs in solution produces an initial IP ensemble, {D^+^A^–^}^‡^, which rapidly relaxes in parallel into fluorescent TIPs and nonfluorescent LIPs, each undergoing CR at different rates. During the initial relaxation, all IPs are detected in both TRFL and TA measurements. After relaxation, TIPs continue to be observed by both TRFL and TA, whereas nonfluorescent LIPs are detected only by TA. In systems where CR is much faster than TIP↔LIP interconversion, such as those examined here, equilibrium is not established, and distinct τ_F_ and τ_A_ are observed. In this case, τ_F_ reflects the CR of TIPs, whereas τ_A_ originates primarily from the CR of LIPs. In systems where CR is slower than or comparable to TIP↔LIP interconversion, equilibrium can be established, yielding similar lifetimes measured by TRFL and TA as reported previously.^22–24^

This revised concurrent mechanism is justified by two general features of the CT excitation of DACs. The first is that CT excitation of DACs typically produces large vibrational (internal) and solvent reorganization energies.^16,17,64–66^ The former arises from internal geometric changes from DA to D^+^A^–^, and the latter arises from the solvent response to the large dipole-moment change. A conservative estimate of the excited-state reorganization energy is half of the steady-state fluorescence Stokes shift,^67–69^ which is 12700/2 = 6350 cm^–1^ for BZ–TCNE in CH_2_Cl_2_.^37^ Because the solvation of ground-state DACs is very limited, the reorganization energy deposited in the initial IPs is expected to be even larger. A reasonable estimate of the vibrational, or ground-state, reorganization energy is half of the steady-state fluorescence Stokes shift for BZ–TCNE in cyclohexane,^69^ which is 8000/2 = 4000 cm^–1^.^37^ Therefore, upon CT excitation, the excited-state reorganization energy for BZ–TCNE in CH_2_Cl_2_ can be as large as ∼1 eV. Within the first few picoseconds, this excess energy is rapidly redistributed into a local heat sink shared by low-frequency interionic modes of the IP and the surrounding first solvent shell (FSS).^70–74^ Before slower energy flow into the bulk solvent occurs (∼10 ps),^35,37^ the locally hot IP/FSS assembly and the floppy nature of the IP allow the system to sample a broad region of configuration space, including LIPs that are separated from TIPs by substantial barriers. Subsequent vibrational cooling then traps the system in these species. These vibrational relaxation and cooling processes are reflected in the early time spectral evolutions observed in our TRFL and visible TA measurements.

The second general feature of CT excitation that supports our proposal is the broad configurational distribution of the initial IPs. DACs in room-temperature solutions are known to undergo large-amplitude intermolecular vibrational motions (),^37,75,76^ and “random pairs” with a wide distribution of D–A separation have also been proposed.^77^ Recent MD simulation studies reveal that DACs do not adopt a single well-defined structure but instead exist as a broad distribution of geometries in solution at room temperature.^35,36,78,79^ Although initial CT excitation may favor DAC structures with sufficient electronic coupling, excitation of other configurations with nonzero oscillator strengths can remain statistically competitive. Consequently, the initial IP ensemble contains a broad distribution of mutual orientations and separations between D^+^ and A^–^,^35,36,78,79^ and an important fraction of initial IPs may possess configurations that facilitate rapid relaxation to LIPs. We note that this second feature bears some similarity to the simultaneous generation of different types of IPs proposed previously by Mohammed and Vauthey to explain the biexponential decay observed in TA measurements of the methylperylene–TCNE complex under CT excitation in polar solvents.^32^

Our experimental results do not provide direct structural information about TIPs and LIPs. Nevertheless, the stronger electronic coupling in TIPs implies a more intimate contact between the two moieties to allow substantial overlap of their frontier MOs; whereas the weak coupling in LIPs suggests structures that hinder such overlap, due to either specific orientations or larger separations between D^+^ and A^–^. The observation of distinct lifetimes further implies that TIPs and LIPs are separated by substantial barriers, such that interconversion is significantly slower than their individual decay rates.

In the following, we discuss possible IP structures based on theoretical considerations. In our previous studies of BZ–TCNE complexes,^37,38^ TDDFT calculations predicted that the lowest singlet CT state (CT_1_) adopts a distorted face-to-face configuration, in which TCNE is laterally shifted from the center of BZ. At this structure, the CT_1_ state exhibits a small but non-negligible fluorescence oscillator strength and can therefore be assigned to the fluorescent TIP. In contrast, recent MD simulations and TRIR anisotropy measurements reported by Rumble and Vauthey suggest a dominant edge-to-face T-shaped IP configuration.^35^ In light of their findings, we have reinvestigated CT_1_-state structures of BZ–TCNE and other DACs using TDDFT/PCM methods with particular attention focused on locating stable geometries other than the face-to-face configuration. Computation details are listed in SI section S6, and the results are summarized in Table 2. It should be emphasized that the TDDFT/PCM results are intended to provide qualitative structural insight rather than definitive structural assignments.

**Table 2:** CT-State Energies, Oscillator Strengths, and Dipole Moments of DACs Calculated at the ωB97XD/aug-cc-pVDZ/PCM Level of Theory

	BZ–TCNE in CH_2_Cl_2_			BZ–TCNE in CCl_4_			TL–TCNE in CH_2_Cl_2_			FB–TCNE in CH_2_Cl_2_		
states at specified structure	VEE^a^ (*f*)^b^	Δ*E* ^c^	μ^d^	VEE^a^ (*f*)^b^	Δ*E* ^c^	μ^d^	VEE^a^ (*f*)^b^	Δ*E* ^c^	μ^d^	VEE^a^ (*f*)^b^	Δ*E* ^c^	μ^d^
S_0_ min.		0.00	1.40		0.00	1.54		0.00	1.81		0.00	2.22
CT_1_ at S_0_ min.	2.95 (0.0000)	2.95	14.7	2.95 (0.0000)	2.95	14.8	2.74 (0.1016)	2.74	13.6	2.91 (0.0635)	2.91	
CT_2_ at S_0_ min.	3.02 (0.0896)	3.02		3.02 (0.0898)	3.02		2.91 (0.0007)	2.91		3.21 (0.0196)	3.21	
CT_1_ at face-to-face CT_1_ min.	2.04 (0.0064)	2.55	13.4	2.02 (0.0036)	2.54	12.1	1.72 (0.0034)	2.22	13.5	1.88 (0.0042)	2.41	14.2
CT_2_ at face-to-face CT_1_ min.	2.71 (0.0357)	3.22		2.68 (0.0235)	3.21		2.63 (0.0317)	3.13		2.90 (0.0296)	3.43	
CT_1_ at T-shaped CT_1_ min.	2.12 (0.0000)	3.00	19.9	2.01 (0.0000)	2.90	18.4						
CT_2_ at T-shaped CT_1_ min.	2.96 (0.0383)	3.84		2.86 (0.0270)	3.76							

a Vertical excitation energy (in eV) of CT states referenced to S_0_ state at the specified structure.

b Oscillator strength of the vertical excitation transition from S_0_ at the specified structure.

c Relative energy (in eV) with respect to S_0_ minimum.

d Dipole moment in Debye.

For BZ–TCNE, we identified a CT_1_-state local minimum corresponding to a T-shaped geometry (Figure S9b) similar to that described by Rumble and Vauthey.^35^ However, at the level of theory used here, this T-shaped structure is predicted to lie ∼0.35–0.45 eV higher in energy than the distorted face-to-face global minimum (Figure 5a and Table 2). In contrast, MD simulations predict a dominant T-shaped configuration.^35^ It is likely that each method favors one structure over the other. The T-shaped IP exhibits a much larger dipole moment (Table 2), and the TDDFT/PCM approach may underestimate its solvation stabilization. Conversely, the Coulombic electrostatic potentials employed in MD simulations may be more appropriate for larger D–A separations in T-shaped configurations, but they may not adequately capture the noncovalent interactions in face-to-face structures, where frontier MO overlap is substantial. These considerations suggest that the T-shaped and distorted face-to-face CT_1_-state structures may be of comparable stability and are separated by a substantial barrier.

**Figure 5:**
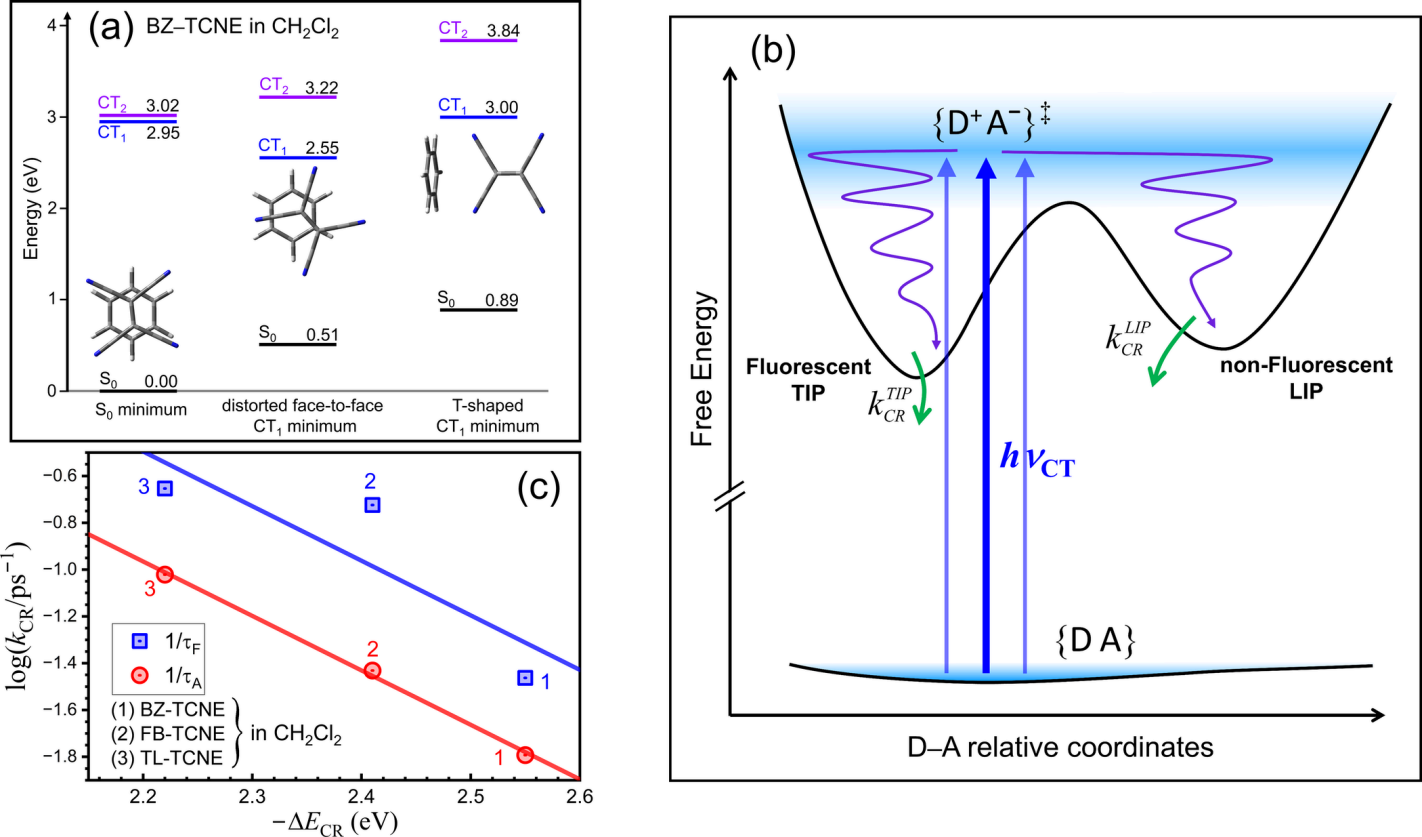
(a) Schematic energy-level diagram showing the relative energies of the S_0_, CT_1_, and CT_2_ states of BZ–TCNE in CH_2_Cl_2_ at three relevant optimized geometries, as described in the text. (b) Schematic free-energy profiles of relevant species involved in CT excitation of DACs and the concurrent parallel relaxation pathways proposed in this work. (c) Plot of the logarithm of CR rates, derived from 1/τ_F_ and 1/τ_A_, against the calculated CT_1_–S_0_ adiabatic energy gap. The straight lines are linear fits to the data and are intended only to guide the eye.

At the C_2V_ T-shaped geometry, the CT_1_–S_0_ optical transition is forbidden by symmetry. The second singlet CT state (CT_2_), which carries a large oscillator strength (Table 2), is predicted to lie at a much higher energy, making CT_1_–CT_2_ vibronic coupling inefficient. As such, the T-shaped CT_1_ state is likely nonfluorescent and only weakly coupled electronically, rendering it a plausible candidate for nonfluorescent LIPs. Although CT excitation may initially favor face-to-face-like structures, the two general features of CT excitation described above allow access to T-shaped structures that can be trapped following vibrational cooling, as illustrated in Figure 5b.

For TL–TCNE and FB–TCNE, TDDFT/PCM calculations predict that CT_1_-state T-shaped structures are not stable and that distorted face-to-face geometries represent the global minima (Figure S11). Nevertheless, a recent MD simulation study of TCNE complexes with a monosubstituted benzene donor, anisole, also predicts a dominant T-shaped IP configuration,^36^ suggesting that TL^+^–TCNE^–^ and FB^+^–TCNE^–^ may likewise exist in T-shaped configurations. It is possible that specific solvation effects and dynamical friction of the solvent cage, which are not included in TDDFT/PCM calculations, contribute to the stabilization of such a metastable T-shaped configuration.

The second possible form of LIPs is SSIPs, as is commonly assumed.^11,15–25^ However, our observations for BZ–TCNE in CCl_4_ appear to disfavor this assignment because SSIPs are not expected to be stabilized in nonpolar solvents. Moreover, MD simulations for the BZ–TCNE IP in CH_2_Cl_2_ did not support the presence of SSIPs.^35^ These findings imply that the LIPs observed here may differ from those produced in bimolecular photoinduced electron transfer over long distances. On the other hand, numerous studies have indicated the formation of SSIPs upon CT excitation of related DCA systems,^11,17–24^ and therefore we cannot completely exclude SSIP contributions. The two general features of CT excitation discussed above may allow access to SSIPs that cannot be reached from relaxed TIPs.

A few points can be briefly elaborated here to further support our interpretation. According to the proposed mechanism, the TA spectra should also contain contributions from TIPs, consistent with components with time constants close to τ_F_ from global analyses. The relatively small amplitudes of the TIP-associated components, compared to those of the LIPs for BZ–TCNE in CH_2_Cl_2_ and CCl_4_, may be partly due to stronger absorption by the TCNE anion in LIPs than in TIPs. This proposition also explains why the components with time constants close to τ_F_ in the TL–TCNE and FB–TCNE TA spectra mostly appear as rising features (negative DAS), because in these two cases, LIP formation competes with the vibrational nonequilibrium CR of TIPs.

Additional support comes from the plot in Figure 5c, where the logarithm of the CR rates for the three DAC systems in CH_2_Cl_2_ is plotted against the reaction driving force, approximated here by the calculated CT_1_–S_0_ adiabatic energy gap (Table 2). The data points corresponding to τ_F_ and τ_A_ follow separate correlations with the driving force, indicating that these lifetimes arise from distinct types of IPs with different electronic couplings.

The mechanism can be readily extended to ternary IPs, (BZ)_2_^+^–TCNE^–^, as shown in Figure 3e–h. In this case, the initial excitation predominantly generates binary IPs, BZ^+^–TCNE^–^, and ternary IPs are subsequently produced by secondary formation through diffusion.^38^ Once binary nonfluorescent LIPs are formed following the initial parallel relaxation, ternary LIPs can be produced through the slower secondary process (∼27 ps), exhibiting CR dynamics slower than those of ternary TIPs.

Finally, we note that the present work is limited to medium-polar and nonpolar solvents of low viscosity. In highly polar media, CR may become competitive with or even faster than the initial parallel relaxation, reducing the branching into LIPs. Likewise, in much more viscous solvents, a more rigid solvent cage would restrict sampling of the configuration space and could also suppress LIP formation.

In conclusion, our combined TRFL and TA studies provide experimental data that support a revised concurrent mechanism following CT excitation of DACs. In contrast to the conventional view, optical excitation of DACs within their CT absorption bands does not initially yield only emissive TIPs; rather, it produces a locally hot and structurally diverse initial IP ensemble that bifurcates into parallel relaxation pathways, leading to fluorescent TIPs and nonfluorescent LIPs, whose independent CR dynamics account for the distinct lifetimes observed in TRFL and TA measurements. This revised mechanism explains why TRFL lifetimes (τ_F_) are systematically shorter than TA lifetimes (τ_A_) in the DAC systems studied here and is important for the correct interpretation of ultrafast spectroscopic data obtained via direct CT excitation of DACs.

## Supplementary Material





## Notes

Experimental setups and details; supplementary experimental data, including steady-state absorption spectra, enlarged views of selected spectral regions, TRFL and TA spectra of a covalently bonded electron donor–acceptor molecule; decay associated spectra obtained from global analyses, quantum chemistry computation methods; optimized molecular structures; tabulated computational results and Cartesian coordinates of optimized molecular structures. (PDF)
Transparent Peer Review report available (PDF)
