# Supplementary material for: Concurrent Generation of Tight and Loose Ion Pairs upon Charge-Transfer Excitation of Electron Donor–Acceptor Complexes in Solution
Source: J Phys Chem Lett. 2026 Jan 26;17(5):1436–46. doi: 10.1021/acs.jpclett.5c03709 (PMC12884513; doi:10.1021/acs.jpclett.5c03709)
Supplement: Supplementary file 1 [file jz5c03709_si_001.pdf]

Supporting Information

Concurrent Generation of Tight and Loose Ion Pairs upon Charge-Transfer Excitation of Electron Donor-Acceptor Complexes in Solution

Guan-Yu Chen (陳冠宇), Yi-Kai Liao(廖翊凱), Pin-Hsun Chen(陳品勳), Yu-Cheng Hsu(許友誠), Pei-Chen Chiang(江沛辰), Yu-Chen Hsu(徐宇辰), Bo-Yu Chang(張博宇), Guan-Sho Chen(陳冠劭), Yi-Fan Wen(溫逸凡), Yu-Fang Yeh(葉友芳), Wen-Teng Hsu (許文騰), Chih-Chang Hung(洪志昌), Chih-Chung Chiu(邱志忠), and Po-Yuan Cheng*(鄭博元)

Department of Chemistry, National Tsing Hua University, Hsinchu, Taiwan, Republic of China

*Corresponding author, Email: pycheng@mx.nthu.edu.tw

Contents **page**

S1. Experimental Methods S3
S1.1. Ultrafast broadband time-resolved fluorescence (TRFL) spectroscopy S4
S1.2. Ultrafast broadband visible transient absorption (TA) spectroscopy S5
S1.3. Sample solutions S7
S1.4. Steady-state absorption spectra S8
S2. Details of SE and GSB bands of TA spectra S9
S3. Semi-log plots of TA and TRFL signals S10
S4. Decay-associated spectra resolved from global fittings S11
S5. TRFL and TA spectra of a covalently-bonded electron donor-acceptor molecule S12
S6. Quantum chemical computations S14
S6.1. Ground-state optimized structures S14
S6.2. CT-state vertical excitation energies and properties S15
S6.3. CT1-state optimized structures S16

List of Figures **page**

Figure S1.. S8

Figure S2.. S9

Figure S3.. S9

Figure S4.. S10

Figure S5.. S11

Figure S6.. S12

Figure S7.. S15

Figure S8.. S18

Figure S9.. S19

Figure S10.. S20

Figure S11.. S21

List of Tables **page**

Table S1.. S7

Table S2.. S17

Table S3.. S22

Table S4.. S22

Table S5.. S23

Table S6.. S24

Table S7.. S24

Table S8.. S25

Table S9.. S26

S1. Experimental Methods

Two types of ultrafast spectroscopic measurements were conducted in this study, namely the ultrafast time-resolved broadband fluorescence (TRFL) and visible-light transient absorption (vis-TA). In both experiments, laser pulses were derived from a femtosecond laser system consisting of a self-mode-locked Ti:sapphire laser (Spectra Physics, Tsunami) and a 1 kHz chirped-pulse regenerative amplifier (CPA; Spectra Physics, Spitfire). A fraction of the CPA output (766–830 nm, ~120 fs FWHM) was frequency doubled to produce excitation pulses at 383–415 nm. Alternatively, the major portion of the CPA output at 800 nm was used to pump an optical parametric amplifier (OPA; Light Conversion, TOPAS-C), and the resulting idler output at 1640 nm was successively frequency-doubled in two thin BBO crystals to produce excitation pulses at 410 nm. The remaining portion of the CPA output was used as the gate pulse in TRFL experiments or as the probe pulse for white-light continuum (WLC) generation in vis-TA experiments.

Details of TRFL and TA experimental setups and procedures are provided in the following sections. In both TRFL and TA measurements, sample solutions were placed in a two-inch-diameter rotating cell with an internal path length of 0.5 or 1 mm, depending on the DAC system. Because only a limited amount of sample solution was available in the cell, data were collected over the shortest timespan possible to minimize potential interference from photoproducts. Extended data-collection periods led to reduced signal intensity and/or additional spectral or temporal features that can be attributed to photoproduct formation. In both TRFL and TA experiments, data from each individual scan were saved for later inspection to monitor potential sample degradation. By inspecting results of each scan, only data acquired before the onset of photo-induced degradation were extracted and averaged for further analysis.

Although short data-acquisition times employed here effectively minimized photoproduct interference, they also inevitably limited the signal-to-noise (S/N) ratio. Therefore, in some TA experiments, data obtained from multiple back-to-back runs using fresh sample solutions were averaged to improve S/N ratio.

S1.1 Ultrafast broadband time-resolved fluorescence (TRFL) spectroscopy

The home-built ultrafast broadband time-resolved fluorescence (TRFL) spectrometer used in this work is based on the optical Kerr gating technique.[1-3] The Kerr shutter consists of a Kerr medium (0.5 mm thick liquid benzene) placed between a pair of crossed wire-grid polarizers (Moxtek, PPL04C). The excitation pulse is focused onto sample solutions contained in a 2”-diameter rotating cell with an internal path length of 0.5 mm (or 1 mm) between two 1 mm-thick fused silica window. A half-wave plate is used to rotate the excitation-laser polarization at the “magic angle” with respect to the first polarizer of the Kerr shutter. The fluorescence is collected by a 90° off-axis 2”-diameter parabolic mirror (effective *f*=75 mm) and is directed through the first polarizer and focused on the Kerr medium by a second 90° off-axis parabolic mirror (effective *f*=150 mm). The gate pulse is sent through a computer-controlled delay line and then steered and focused to spatially overlap with the fluorescence spot at the Kerr medium. The polarization of the gate pulse was rotated by 45° with respect to the first polarizer to maximize the gating efficiency.[1-3] Typical pulse energies are ~1-2 μJ/pulse for excitation and ~35-70 μJ/pulse for gating.

The gated fluorescence is collected and imaged onto the entrance slit of a spectrograph (Zolix, Omni-λ 300, 150 lines/mm grating) via a pair of achromatic lenses. The entrance slit is adjusted to give an effective bandpass of about 6 nm. Long-pass and short-pass filters with appropriate cutoff wavelengths are placed in front of the spectrograph entrance slit to reject scattered light from the excitation pulse and its fundamental. The dispersed fluorescence spectra are recorded by a TE-cooled CCD camera (Andor, DU970N-BV). In the TRFL experiments presented here, the combination of CCD camera binning and the spectrograph bandpass yields an effective spectral resolution of ~10 nm as estimated from solvent Raman lines.

A computer program was devised to automate data acquisition. The program moves the translation stage to a series of preselected positions and records the gated fluorescence spectra at the corresponding delay times. A background spectrum obtained at a negative delay time (typically −20 ps) at the beginning of each scan is subtracted from spectra measured at other delay times. The TRFL spectra at each delay time are accumulated and averaged by repeatedly scanning the translation stage back and forth until a satisfactory signal-to-noise ratio is achieved.

All TRFL spectra presented here are corrected for spectral sensitivity and temporal dispersion using procedures described in our previous report.[4]

The temporal instrument response function (IRF) of the system is obtained by measuring the Raman response of a pure solvent. The measured full width at half maximum (FWHM) of IRF depends on the pathlength of the Kerr medium (liquid benzene) used for each system. Typically, liquid benzene placed in a cell of 0.5 mm internal pathlength was used as the Kerr medium, which gives an IRF of about 0.35 ps in width. For systems with lower fluorescence intensity, a 1 mm liquid benzene pathlength is used to increase the gating efficiency. which degrades the temporal resolution to about 0.5 ps. In experiments where higher temporal resolutions is required, a 1 mm-thick fused silica plate can be used as the Kerr medium, providing a better temporal resolution of about 0.21 ps at the cost of gating efficiency. All TRFL measurements reported in this work were carried out with an effective temporal IRF of about 0.35–0.5 ps in width.

S1.2 Ultrafast broadband visible transient absorption (TA) spectroscopy

The home-built ultrafast visible broadband TA spectrometer used in this work employs a dual-beam configuration with single-shot spectral referencing. The pump pulses are produced in the same manner as in the TRFL experiments. Typical excitation pulse energies used in TA measurements are ~1-2 μJ/pulse. A small fraction of the CPA output is directed through a computer-controlled optical delay line and then focused into a 3-mm cell containing pure water to produce the white-light continuum (WLC) pulse. The WLC pulse is recollimated with a 90° parabolic mirror and sent into a 1 mm pathlength cuvette containing an aqueous $CuSO_4$ solution (~0.75 M), which acts as an optical filter to reduce the residual fundamental intensity. After passing through a thin wire-grid polarizer, the resulting WLC pulse is split into probe and reference pulses via an optical wedge.

The probe and reference pulses are focused at the sample cell and directed into a pair of fiber-coupled photodiode array spectrometers (Avantes, AvaSpec-128, 300 l/mm grating, 50 μm slit, effective bandpass≈6 nm) using two identical sets of off-axis parabolic mirrors. The pump pulse was focused at ~2 cm behind the sample cell with an f= 500 mm CaF_2 lens and spatially overlapped with the probe pulse at the sample cell. The pump polarization is set at the "magic angle" with respect to that of the WLC probe with a half-wave plate. The two

spectrometers record the WLC spectra of the probe and reference pulses shot by shot, and a synchronized optical chopper (Thorlabs, MC1000) modulates the pump beam so that individual ‘pump-on’ and ‘pump-off’ spectra are recorded separately and stored in an on-board memory for subsequent data transfer. The transient absorption spectrum $\Delta A(\lambda,t)$, i.e., the pump-induced change in the sample absorbance at delay time t, is calculated using the following expression,

$$\Delta \mathrm{A}(\lambda,t) = -\log\left(\frac{I_P^{*}(\lambda,t)/I_R^{*}(\lambda)}{I_P^{\mathrm{d}}(\lambda)/I_R^{\mathrm{d}}(\lambda)}\right), \qquad \text{Eq. (S1)}$$

, where I_P (λ) and I_R (λ) are the probe and reference spectra, respectively, and the superscripts denote that the pump-on (*) and pump-off (°) shots. The optical chopper is typically operated at 500 Hz to block every-other pump pulse to facilitate single-shot referencing. At each preselected delay time in a scan, TA spectra are calculated from consecutively acquired pump-on/off shots and averaged over 1000-2000 shots. Alternatively, the optical chopper can be run at 250 Hz and the spectrometers set to integrate two adjacent shots for the pump-on and pump-off spectra. In this double-shot referencing scheme, the time required to transfer data from the on-board memory to computer is reduced by a factor of two, with a slight trade-off in S/N ratio.

A background pure-solvent response is recorded under the same conditions immediately after each TA measurement of a sample solution, and transient ‘artifacts’ due to various nonlinear processes are removed by subtracting this background signal using the procedure suggested by Lorenc et al.[5]

To correct for temporal group delay dispersion, we first measured the wavelength-dependent time-zero curve of the probe WLC pulse with respect to the pump pulse using optical Kerr gating. To this end, a second wire-grid polarizer is placed in the probe beam path after the rotating cell, with its transmission axis rotated at 90° relative to that of the first polarizer. A pure solvent loaded in the same pathlength cell is used as a Kerr medium, and the excitation pulse serves as the gating pulse. By monitoring the gated WLC signal, the time zero at each probe wavelength can be determined under the same conditions as in the TA experiment. To correct the temporal dispersion in the TA data, the TA time traces at each probe wavelength are interpolated using a cubic spline algorithm and are then time-shifted according to the corresponding time-zero dispersion curve. The temporal IRF of the system is measured in the same manner by replacing the sample cell with a 1mm-thick fused silica plate to act as the Kerr medium. The FWHM of the measured temporal IRF is about 0.2 ps.

S1.3 Sample solutions

Five DAC systems are studied in this work. Concentrations (or mole fractions) of donors and acceptor (TCNE) of these DAC solutions are listed in Table **S1**. TCNE was purchased from Aldrich (98 %) and was sublimed twice before use. Benzene (Merck, Uvasol grade), Toluene (Merck, Uvasol grad, >99.8%), Fluorobenzene (TCI, >99%), and CH_2Cl_2 (Baker, PHOTREX grad, 99.9 %) were used as received, while CCl_4 (Showa, 99.5 %) was fractionally distilled under dry nitrogen before use. Sample solutions were freshly prepared before each run of experiment. All experiments were carried out at room temperature of ~23 °C.

Except for the BZ/TCNE in 10% BZ/CCl_4 solution, all other solutions were prepared to ensure that 1:1 binary D–TCNE complexes dominate in both the ground and excited states.[6,7] In some cases, the donor concentration was reduced to confirm that the observed dynamics remained changed. In TA experiments, the absence of the characteristic charge-resonance absorption band of the aromatic dimer cation provides a strong support for the predominance of the 1:1 binary CT state.

Table S1. Concentrations of donors and acceptor (TCNE) in solution and excitation wavelengths used in the five DAC systems studied in the TRFL and TA measurements

Solutions used in TRFL measurements				
DAC system	TCNE molarity	donor molarity (x_{donor})[a]	solvent	λ_{exc}/nm
BZ–TCNE in CH_2Cl_2	0.02 M	0.2 M	CH_2Cl_2	383
TL–TCNE in CH_2Cl_2	0.025 M	0.25 M	CH_2Cl_2	415
FB–TCNE in CH_2Cl_2	0.032 M	0.32 M	CH_2Cl_2	383
BZ–TCNE in CCl_4	0.0015 M	0.04 M (x_{BZ}=0.35%)	CCl_4	383
BZ/TCNE in 10% BZ/CCl_4[b]	0.0015 M	1.04 M (x_{BZ}=10%)	CCl_4	383
Solutions used in TA measurements				
DAC system	TCNE molarity	donor molarity (x_{donor})[a]	solvent	λ_{exc}/nm
BZ–TCNE in CH_2Cl_2	0.04 M	0.15 M	CH_2Cl_2	383
TL–TCNE in CH_2Cl_2	0.04 M	0.15 M	CH_2Cl_2	410
FB–TCNE in CH_2Cl_2	0.03 M	0.38 M	CH_2Cl_2	390
BZ–TCNE in CCl_4	0.0015 M	0.054 M (x_{BZ}=0.5%)	CCl_4	390
BZ/TCNE in 10% BZ/CCl_4[b]	0.01 M	1.04 M (x_{BZ}=10%)	BZ/CCl_4	390

[a]Corresponding mole fraction of donor in solution. [b]This solution contains binary and ternary

(2:1) complexes.

S1.4 Steady-state absorption spectroscopy

Steady-state absorption spectra of the sample solutions used for TRFL measurements (listed in Table S1) were recorded using a UV–vis absorption spectrometer (Hitachi U-3900). Figure **S1** shows the steady-state absorption spectra of the five DAC systems investigated in this study. The absorption spectra of the BZ–TCNE complex in CH_2Cl_2, CCl_4, and the 10% BZ/CCl_4 cosolvent are very similar, with band maxima located near 390 nm. For the TL–TCNE and FB–TCNE complexes in CH_2Cl_2, the absorption maxima are red- and blue-shifted, respectively, relative to BZ–TCNE, reflecting the influence of donor redox potentials on the CT absorption. In addition, their absorption bands are noticeably broader than those of BZ–TCNE, likely due to overlap between two closely spaced CT transitions.

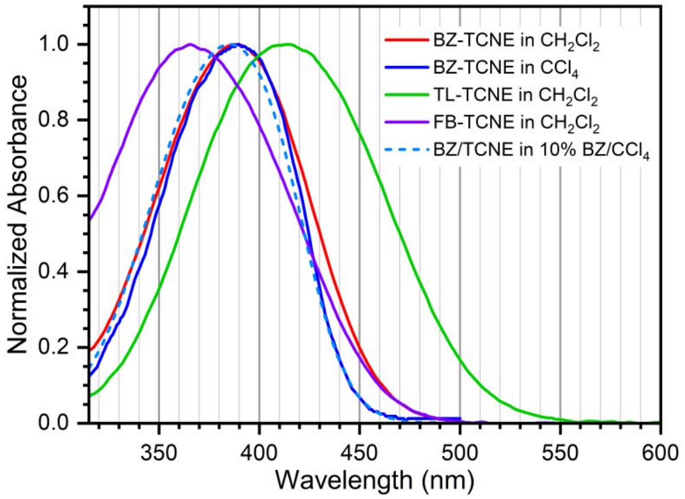

Figure S1. Steady-state absorption spectra of the sample solutions used in this study (Table **S1**, solutions used in TRFL measurements).

S2. Details of SE and GSB bands of TA spectra

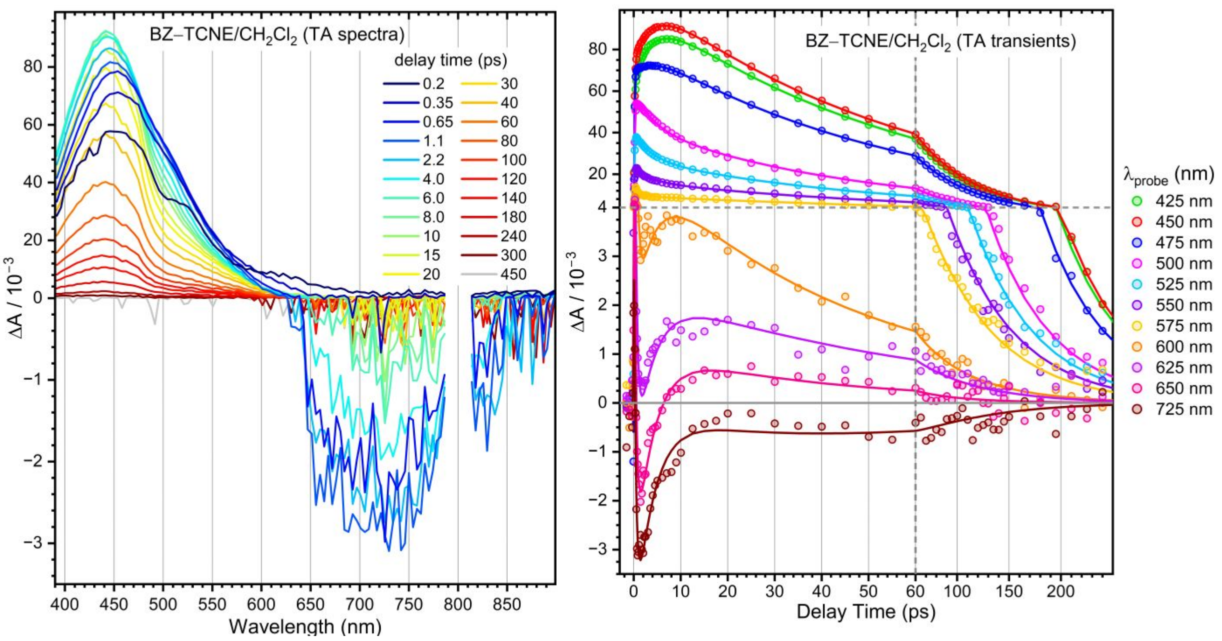

Figure S2. Enlarged views of the stimulated emission (SE) band region in the TA data of BZ–TCNE in CH_2Cl_2 at selected delay times and wavelengths.

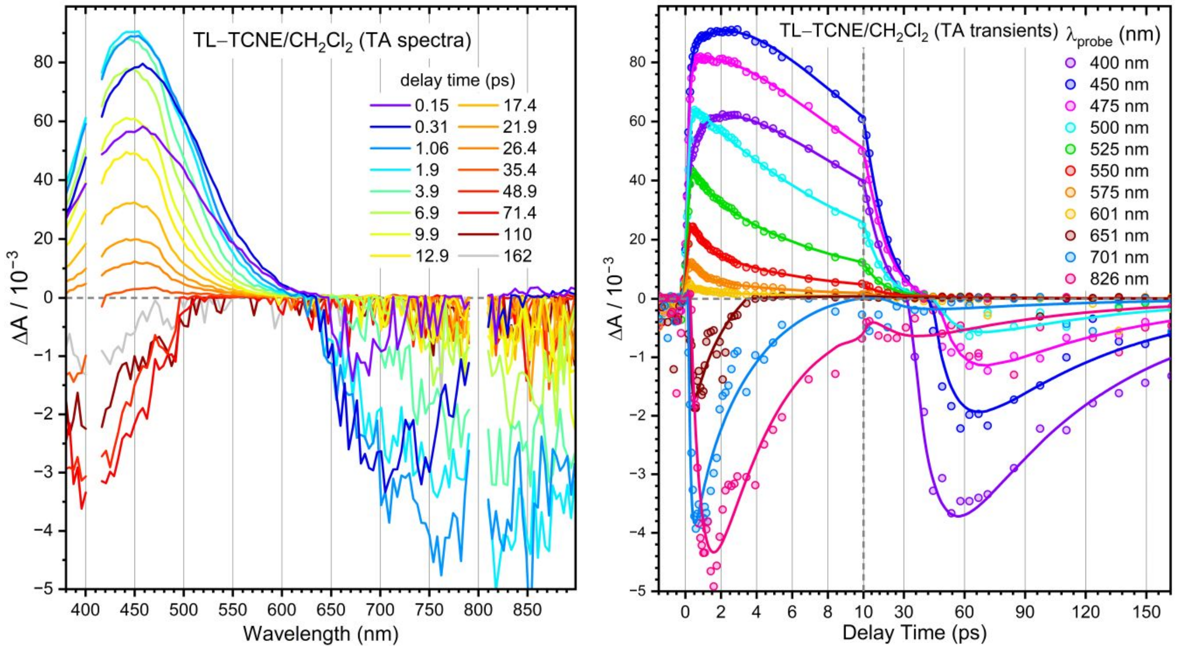

Figure S3. Enlarged views of the SE and ground-state bleach (GSB) regions of the TA data of TL–TCNE in CH_2Cl_2 at selected delay times and wavelengths. The relatively slow GSB recovery (72 ps), compared to CR (τ_A=10.5 ps) and vibrational cooling, may indicate a loose IP configuration prior to CR, although a slow re-equilibration process involving a large excess energy of ~2.2 eV in the flexible ground state cannot be excluded.

S3. Semi-log plots of TRFL and TA signals

The semi-log plots of TRFL and TA signals of BZ-TCNE in CCl_4 shown in Figure **S4** reveal that, beyond ~20 ps, the fluorescence signal decays single-exponentially, while the TA signal contains more than one decay component, supporting the need to include a component fixed at 153 ps (τ_F) in the global fitting of TA spectra.

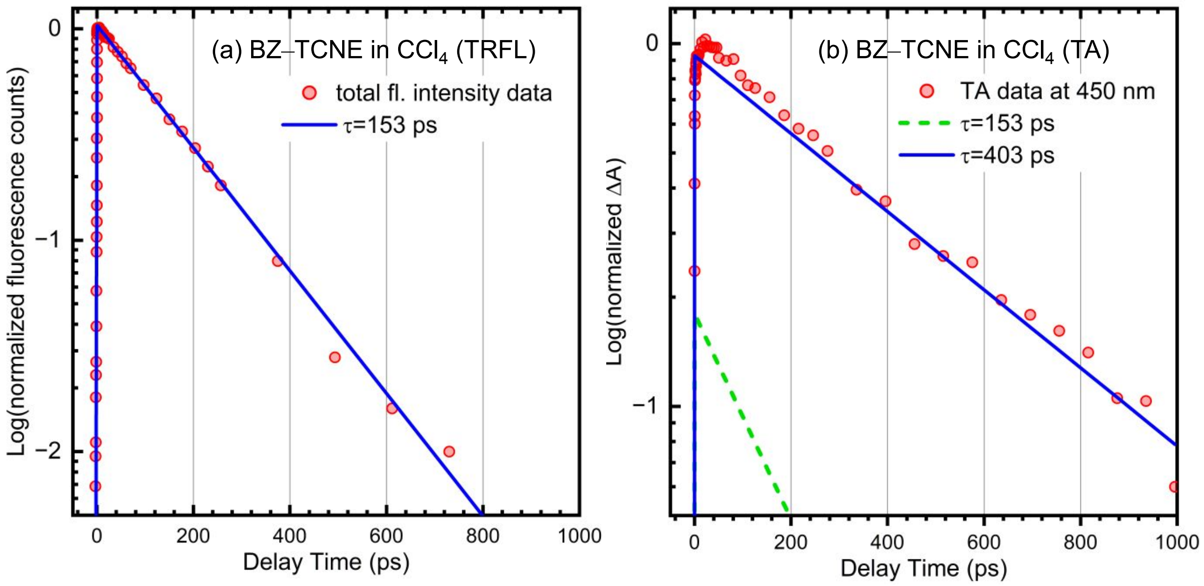

Figure S4. (a) Semi-log plot of the time dependence of total fluorescence intensity derived from the TRFL spectra of BZ–TCNE in CCl_4. Open circles are data points and the solid line is a component with τ=153 ps obtained from a multiexponential fit. (b) Semi-log plot of the TA signal of BZ–TCNE in CCl_4 at 450 nm. Open circles are data points, and the green and blue lines are components with τ=153 ps (fixed) and τ=403 ps, respectively, obtained from a multiexponential fit.

S4. Decay-Associated Spectra Resolved from Global Fittings

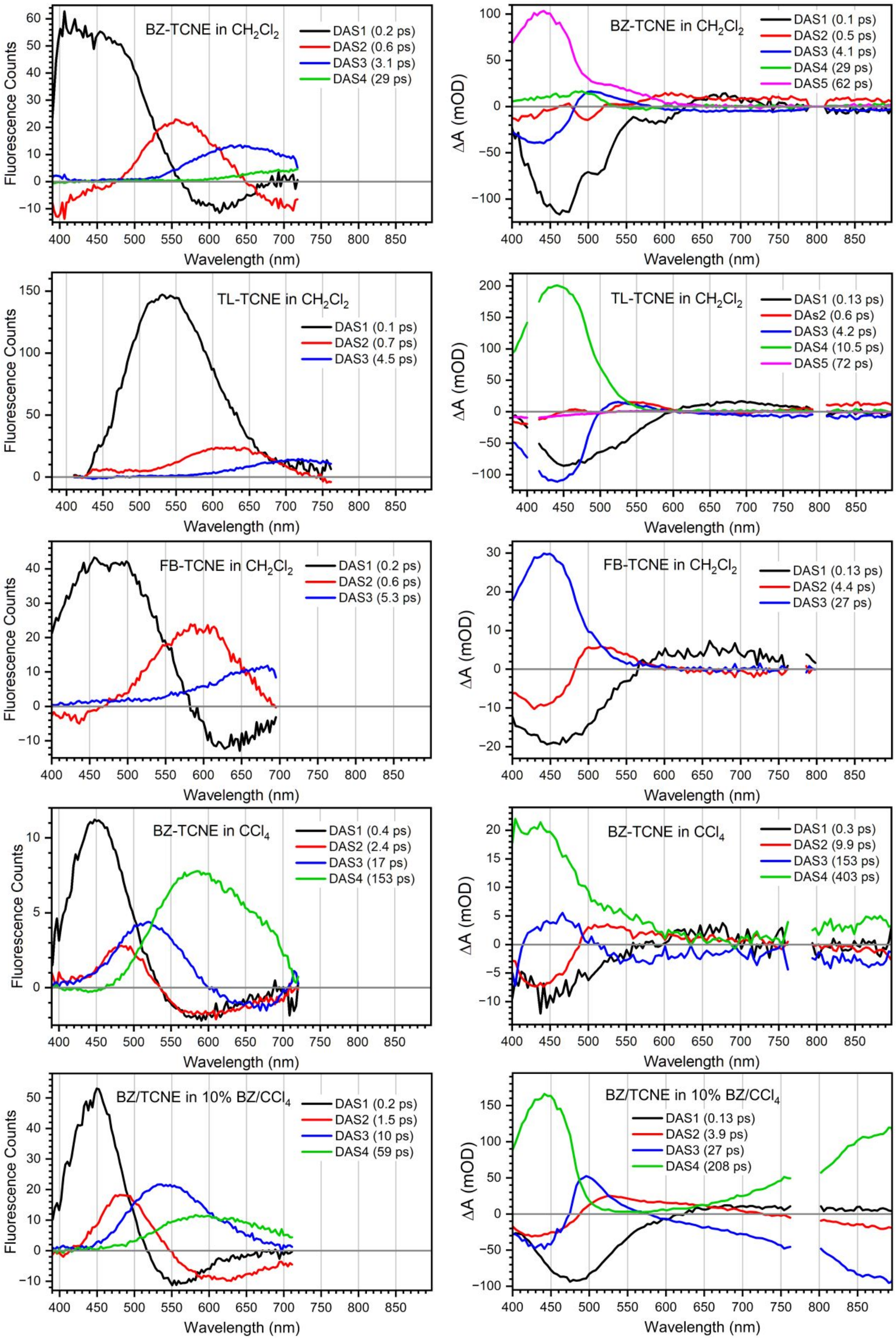

Figure S5. Decay-associated spectra (DAS) obtained from global fits of the TRFL (left) and TA (right) data presented in this work.

S5. TRFL and TA Spectra of a Covalently Bonded Donor-Acceptor dye molecule

In this section, we present ultrafast TRFL and TA spectra of *trans*-4-(*N*,*N*-dimethylamino)-4'-nitrostilbene (DMANS, Figure **S5**(a)), excited into its S_1 state in acetone. The purpose of these supporting experiments is twofold. First, we want to confirm that the discrepancy in lifetimes measured by TRFL and TA reported in this work is not caused by the different experimental setups used in our laboratory for these two techniques. Second, we show that, for a covalently bonded D–A system with a CT-state lifetime comparable to that of BZ–TCNE in CH_2Cl_2, the lifetimes measured by TRFL and TA are indeed nearly identical.

The TRFL and TA spectra of DMANS in acetone are displayed in Figure **S5**. Both TRFL and TA spectra exhibit rapid spectral evolution within the first few picoseconds due to solvation and vibrational/structural relaxation, followed by slower intensity decays. The TA spectra

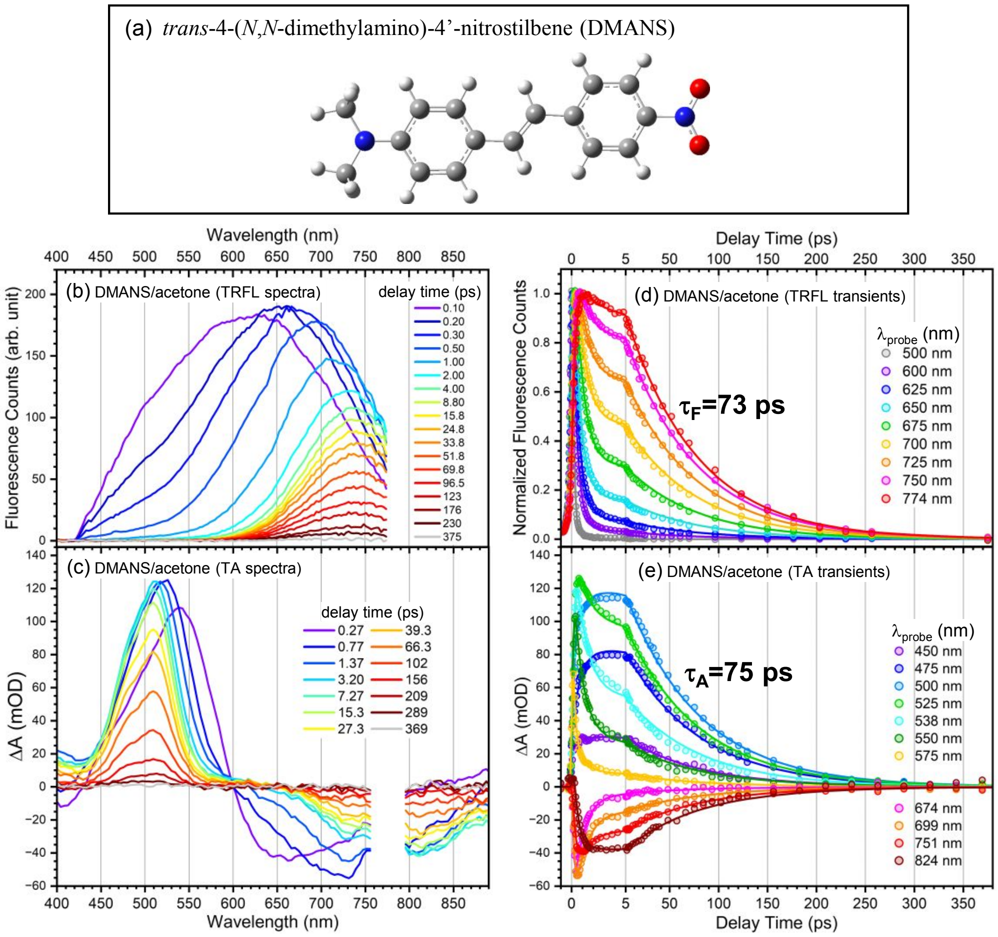

Figure S6. (a) Molecular structure of DMANS. (b) TRFL and (c) TA wavelength spectra of DMANS in acetone at selected delay times. (d) TRFL and (e) TA transients of DMANS in acetone at selected wavelengths; open circles are data points and solid lines are the results of global fits. Excitation wavelengths were 415 and 383 nm for the TRFL and TA experiments, respectively.

contain a distinct ESA band at ~520 nm and a SE band centered at ~750 nm. The temporal evolutions of the SE band are consistent with the TRFL spectra. Global analysis shows that the excited-state lifetimes measured by TRFL (τ_F=73 ps) and TA (τ_A=75 ps) are essentially identical.

DMANS is a representative push-pull charge-transfer dye molecule, in which an electron donor group is covalently linked to an electron acceptor group through a π-conjugated bridge. This stands in contrast to DACs, where separate donor and acceptor molecules are associated only through noncovalent interactions, resulting in far greater structural flexibility. Photoexcitation of DMANS to its S_1 state results in a pronounced intramolecular charge transfer (ICT) and can lead to the formation of twisted intramolecular charge-transfer (TICT) states in polar solvents.[6,8]

Thus, the DMANS results serve as an excellent benchmark for comparison with DACs presented in this work. In both cases, CT excitation leads to rapid spectral evolution within the first few picoseconds in their TRFL and TA spectra, followed by slower intensity decays on similar timescales. However, for the covalently bonded and structurally more rigid DA system (DMANS), the CT-state lifetimes measured by TRFL and TA are nearly identical, as expected when both techniques detect the same transient species (the TICT state). In contrast, for noncovalently bound and structurally flexible DACs studied here, the CT-state lifetimes measured by TRFL and TA differ markedly, suggesting that the two methods detect different transient populations.

Moreover, the DMANS results demonstrate that the lifetime disparities observed in DACs studied in this work are intrinsic to their relaxation dynamics, rather than artifacts arising from differences in TRFL and TA setups.

S6. Quantum Chemical Computations

Density functional theory (DFT) calculations were performed using the ωB97XD functional, which has been shown to provide reliable performance for non-covalently bound molecular complexes[9,10] as well as CT excitation energies with time-dependent (TD) calculations.[11] Solvent effects were included using the polarizable continuum model (PCM) with the integral equation formalism variant[12] All calculations were carried out with the Gaussian 16 program.[13]

S6.1 Ground-state optimized structures

The ground-state (S_0) structures of BZ–TCNE complexes in CH_2Cl_2 and CCl_4 were optimized at the ωB97XD/aug-cc-pVDZ/PCM level of theory. As expected, the optimized ground-state corresponds to a face-to-face configuration with near C_{2V} symmetry, as shown in Figure **S7(a)**. A second optimized structure, in which the mutual orientation between BZ and TCNE is rotated by ~90° (Figure S**7(b)**), lies only slightly higher in energy by ~0.00047 eV (~3.8 cm^{-1}). The existence of these two nearly isoenergetic conformers imply a rather flat potential energy surface along the torsional coordinate about the axis perpendicular to the molecular planes. The Cartesian coordinates of the most stable conformer are listed in Table **S3**.

The lowest seven vibrational frequencies below 100 cm^{-1} correspond to various intermolecular modes between BZ and TCNE (Figure **S10(a)**), indicating that the complex is very structural flexible and can undergo large-amplitude intermolecular vibrational motions at room temperature, sampling a wide range of configurations. This picture is consistent with a recent molecular dynamics (MD) simulation study,[14] which showed that although the face-to-face sandwich geometry is the most probable ground-state conformation, the BZ-TCNE complex does not adopt a well-defined structure and a high degree of structural disorder is present in room-temperature solution.

Ground-state optimized structures of TL–TCNE and FB–TCNE complexes in CH_2Cl_2 were computed in the same manner. The structures of the most stable conformer are shown in Figure **S11**, and their Cartesian coordinates are listed in Tables **S4** and **S5**.

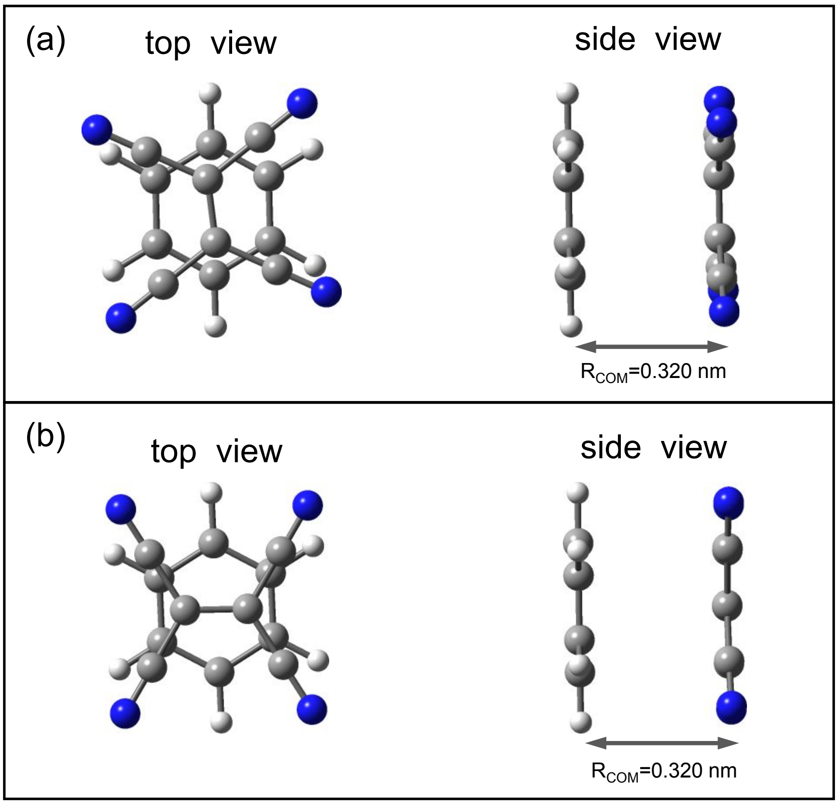

Figure S7. Two nearly isoenergetic conformers of the ground-state BZ–TCNE complex calculated at the ωB97XD/aug-cc-pVDZ/PCM level of theory. Conformer (a) is more stable than conformer (b) by only ~3.8 cm^{-1} in CH_2Cl_2.

S6.2 CT-state vertical excitation energies and properties

Vertical excitation energies of the singlet excited states of the BZ–TCNE complex in CH_2Cl_2 and CCl_4 were calculated at the ground-state optimized structures at the TD-ωB97XD/aug-cc-pVDZ/PCM level of theory. At the Franck-Condon (FC) structures, the first two nearly degenerate singlet excited states, hereafter denoted as CT_1 and CT_2, are separated by merely ~0.07 eV in both solvents (Table 2 and Figure **S8**). They arise predominantly from excitation of electrons from the nearly degenerate HOMO and HOMO−1, which are largely localized on BZ, to the LUMO localized on TCNE.

Upon vertical excitation, the dipole moment of the complex is predicted to increase dramatically from ~1.4 Debye in the ground state to ~14.7 Debye in the CT_1 state (Table **S2**), consistent with the CT nature of the transition. The CT_1-state dipole moment at the FC structure corresponds to an effective charge separation of q=±0.96 e over a center-of-mass distance of 0.320 nm, and the net charges residing on each moiety, evaluated by natural population analysis (NPA), are q=±0.968 e (Table **S2**). These values indicate that the CT_1 state at the FC structure

can indeed be well described as an ion pair.

Under strict C_{2V} symmetry, the S_0→CT_1 transition is symmetry-forbidden, whereas the transition to the CT_2 state carries most of the oscillator strength (Table **2**). However, because of the floppy structure of the BZ−TCNE complex, large-amplitude intermolecular motions at room temperature are expected to induce vibronic coupling, giving the CT_1 state a substantial excitation probability. Our calculations also show that at less symmetric structures the oscillator strengths of the CT_1 and CT_2 excitations can become comparable.

As demonstrated recently, TDDFT calculations performed on representative BZ–TCNE conformations extracted from MD simulations satisfactorily reproduce the experimental steady-state visible absorption spectrum and reveal that it consists of two closely spaced, overlapping broad CT_1 and CT_2 bands.[14] Consequently, excitation near the maximum of the CT band likely excites both CT_1 and CT_2 states with similar probabilities. If initial excitation occurs to the CT_2 state, an ultrafast CT_2→CT_1 internal conversion is expected because of the facile symmetry-breaking intermolecular motions and the very small energy gap. Therefore, subsequent relaxation is expected to occur in the CT_1 state.

Vertical excitation energies of TL–TCNE and FB–TCNE complexes at their ground-state optimized structure in CH_2Cl_2 were computed in the same manner, and the results are listed in Table **2**.

S6.3 CT_1-state optimized structures

The CT_1-state structures of the BZ–TCNE complex were optimized at the TD-ωB97XD/cc-pVDZ/PCM level of theory in CH_2Cl_2 and CCl_4. In this work, particular attention was given to identify stable CT_1-state structures that differ from the face-to-face configuration. Optimization starting from the ground-state geometry resulted in a laterally shifted face-to-face structure in both solvents (Figure **S9** and Table **S6**). On the other hand, optimization initiated from a geometry with a large tilt between the BZ and TCNE molecular planes yielded a T-shaped (edge-to-face) configuration, in which the long edge of the TCNE anion face the BZ molecular plane (Figure **S9** and Table **S7**). Vibrational frequency analyses for these two structures (Figure **S10(b) and (c)**) showed no imaginary frequencies, confirming that they are true minima.

Vertical excitation energies were also calculated at the two CT_1-state optimized structures at the same level of theory. The relative energetics of the S_0, CT_1 and CT_2 states at the three relevant optimized structures are summarized schematically in Figure **S8**. The CT_1-state T-shaped structure is predicted to lie higher in energy than the distorted face-to-face structure by ~0.45 in CH_2Cl_2 and ~0.35 eV in CCl_4 (Figure **S8**). Thus, in the CT_1 state, the distorted face-to-face structure is the global minimum, while the T-shaped configuration corresponds to a local minimum. The very low frequencies of the interionic modes (Figure **S10(c)**) indicate that the T-shaped conformer represents a metastable structure with a shallow potential well. However, specific solvation effects and solvent frictions that are not included in TDDFT/PCM calculations may enhance the stability of the T-shaped structure.

The dipole moment of the T-shaped structure (~19 Debye) is predicted to be significantly greater than that of the distorted face-to-face conformation (~13 Debye), likely due to the increased center-of-mass separation between BZ and TCNE (from ~0.328 nm to ~0.459 nm) in the T-shaped structure. The net charges residing on each moiety, evaluated by NPA, are $q=\pm 0.913\ e$ and $q=\pm 0.868\ e$ for the distorted face-to-face structure and T-shaped structures, respectively (Table **S3**).

CT_1-state optimized structures of the TL–TCNE and FB–TCNE complexes in CH_2Cl_2 were also calculated in the same manner. In both cases, optimization initiated from either T-shaped or face-to-face starting geometries converged to the same laterally shifted face-to-face structure. The optimized CT_1-state structures are shown in Figure **S11**, and their Cartesian coordinates are listed in Tables **S8** and **S9**.

Table S2 Calculated dipole moments, net charges on BZ and TCNE, and distances between center of mass of BZ and TCNE of the CT_1 state of BZ–TCNE in CH_2Cl_2 at three structures

structure	μ (Debye)[a]	q (e)[b]	R_{COM} (nm)[c]
S_0 minimum (FC)	14.7	±0.968	0.320
distorted face-to-face minimum	13.4	±0.913	0.328
T-shaped minimum	19.9	±0.868	0.459

[a]Electric dipole moment in Debye. [b]Net charges residing on BZ and TCNE evaluated by natural population analysis. [c]Distance between the center of mass of BZ and TCNE.

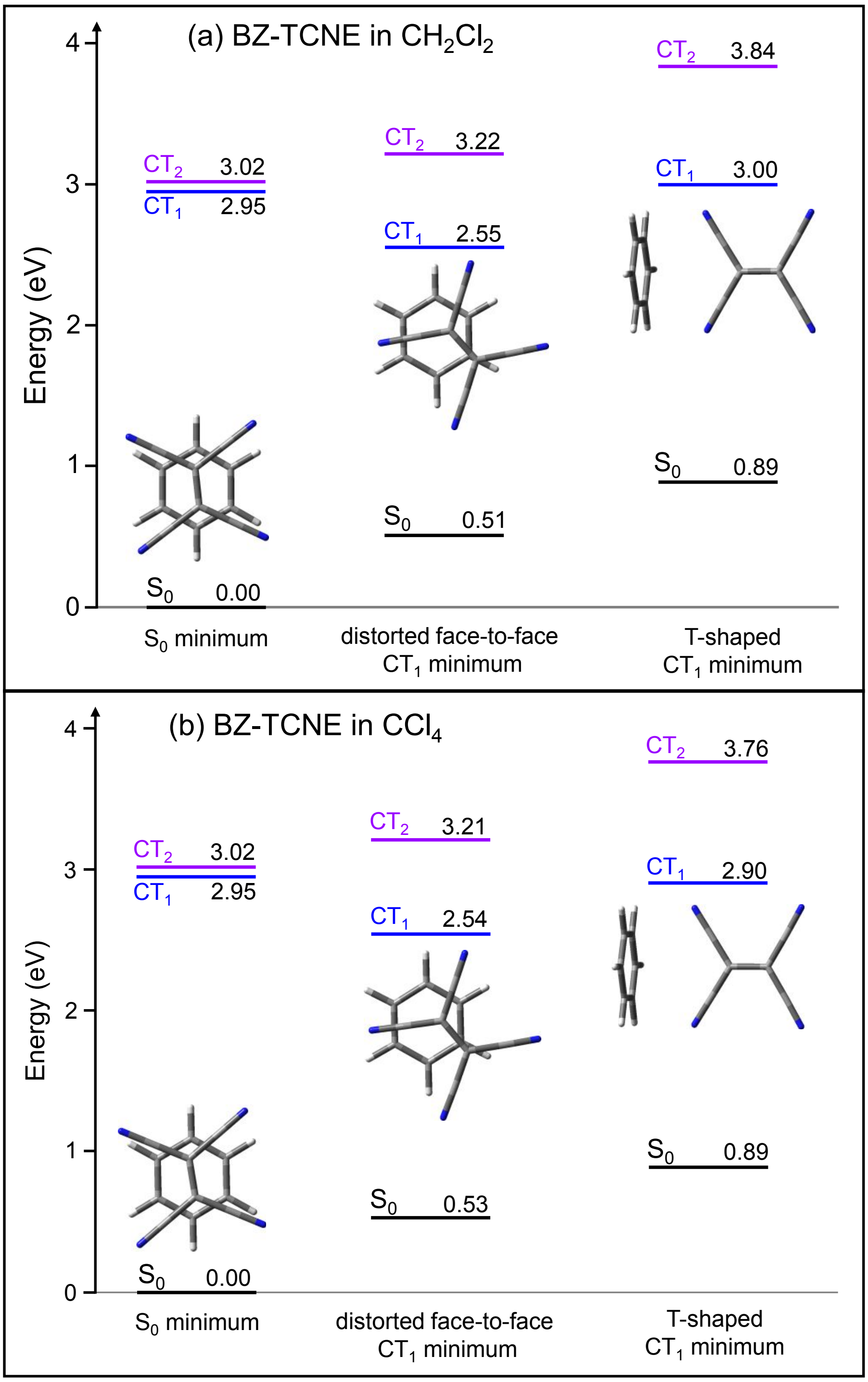

Figure S8. Schematic energy-level diagrams showing the relative energies (in eV) of the S_0, CT_1, and CT_2 states of BZ–TCNE at three optimized geometries, referenced to the S_0-state minimum. (a) in CH_2Cl_2 and (b) in CCl_4. Structures and energies were calculated at the TD-ωB97XD/aug-cc-pVDZ/PCM level of theory.

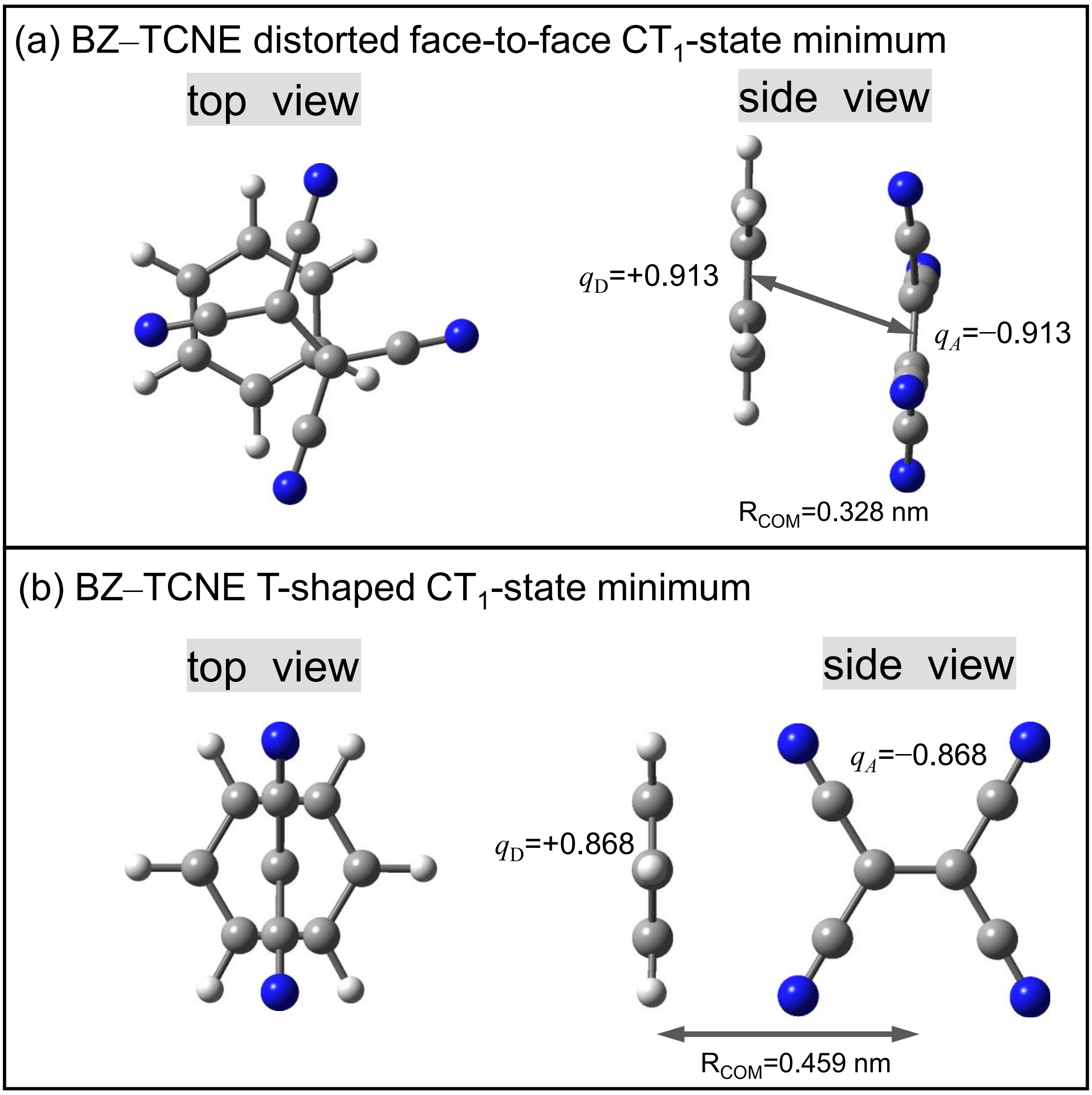

Figure S9. Top and side views of the optimized CT_1-state structures of BZ–TCNE in CH_2Cl_2 for the (a) distorted face-to-face and (b) T-shaped minima, calculated at the ωB97XD/aug-cc-pVDZ/PCM level of theory. The net charges residing on BZ (q_D) and TCNE (q_A) are evaluated by natural population analysis (NPA).

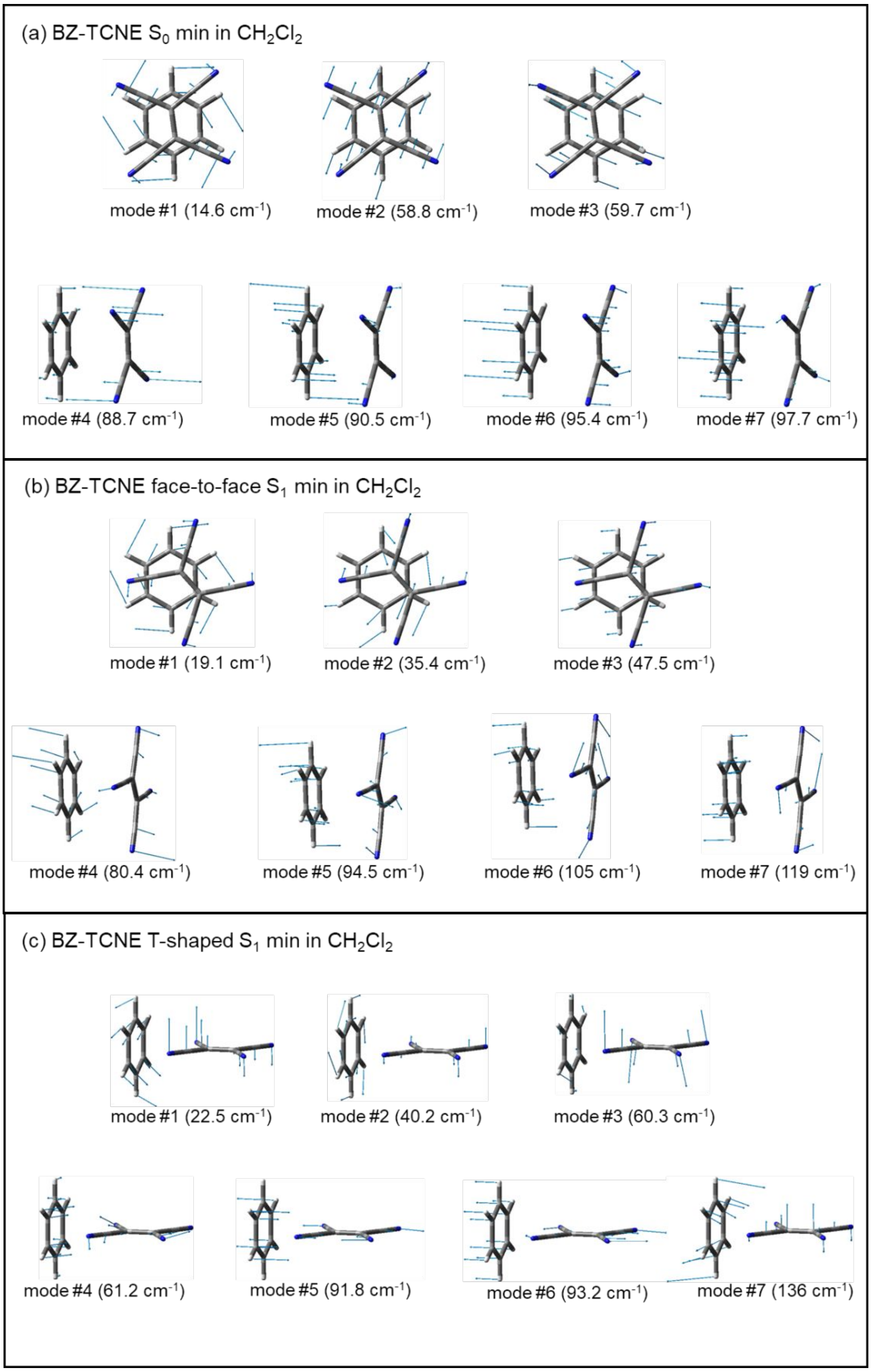

Figure S10. The seven lowest-frequency vibrational modes of the BZ–TCNE complex calculated at the optimized structures of (a) the S_0 ground state, (b) the distorted face-to-face CT_1 state, and (c) the T-shaped CT_1 state at the ωB97XD/aug-cc-pVDZ/PCM level of theory.

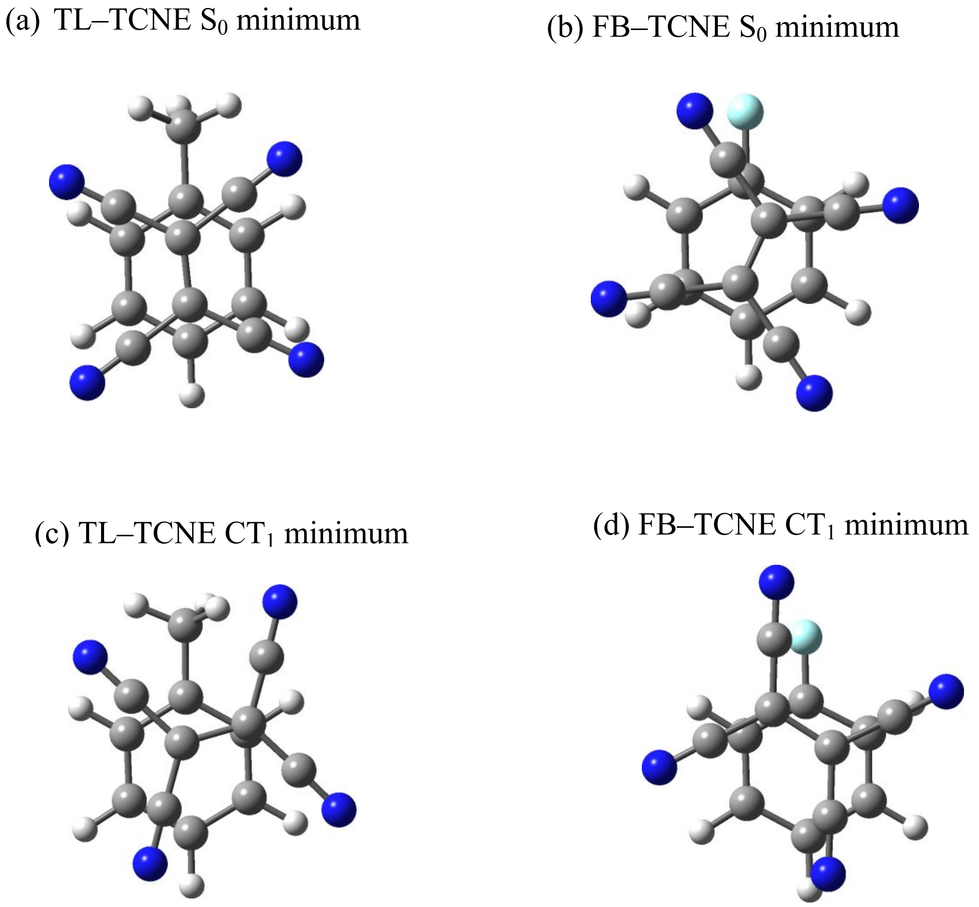

Figure S11. Optimized CT_1-state structures of the of the (a) TL–TCNE and (b) FB–TCNE complexes in CH_2Cl_2 calculated at the ωB97XD/aug-cc-pVDZ/PCM level of theory.

Table S3. Cartesian coordinates of the BZ–TCNE ground-state (S_0) structure optimized at the ωB97XD/aug-cc-pVDZ/PCM level of theory

	in CH_2Cl_2					in CCl_4		
Atom	X	Y	Z		Atom	X	Y	Z
C	-1.2728720	0.0313910	0.6411550		C	-1.2494220	-0.0246300	0.6763520
C	-1.2236250	0.0185110	-0.7161350		C	-1.2464660	-0.0235830	-0.6820970
C	-1.2471500	-1.2134220	-1.4506050		C	-1.2981980	1.2006340	-1.4275260
C	-1.3554380	-1.1860280	1.3952450		C	-1.3001050	1.1987120	1.4233270
C	-1.2879290	1.2635560	1.3751180		C	-1.2469190	-1.2496300	1.4222410
C	-1.1914700	1.2365190	-1.4730210		C	-1.2351160	-1.2474230	-1.4297570
N	-1.2869010	-2.2050670	-2.0455110		N	-1.3630050	2.1834590	-2.0352870
N	-1.4433960	-2.1656300	2.0047340		N	-1.3617050	2.1813000	2.0318350
N	-1.1902550	2.2175040	-2.0865930		N	-1.2484960	-2.2311140	-2.0394270
N	-1.3184800	2.2559030	1.9693740		N	-1.2705050	-2.2336100	2.0311120
C	1.9046300	0.1190010	1.4443640		C	1.9462020	0.2348030	1.3851720
C	1.9590480	1.2429470	0.6161820		C	1.9775410	-1.0619550	0.8656740
C	2.0102600	1.0852880	-0.7690490		C	1.9845880	-1.2593170	-0.5150810
C	2.0025380	-0.1961930	-1.3266760		C	1.9634680	-0.1598330	-1.3762830
C	1.9542150	-1.3201610	-0.4982240		C	1.9393480	1.1370570	-0.8567810
C	1.9071440	-1.1626710	0.8872100		C	1.9292040	1.3343690	0.5240440
H	1.8634760	0.2418250	2.5266300		H	1.9383780	0.3890380	2.4642150
H	1.9578590	2.2414890	1.0520720		H	1.9894480	-1.9184570	1.5389460
H	2.0466900	1.9605860	-1.4167650		H	2.0004890	-2.2698280	-0.9221750
H	2.0364210	-0.3188610	-2.4091760		H	1.9686070	-0.3142850	-2.4553020
H	1.9474990	-2.3184250	-0.9347270		H	1.9217820	1.9934010	-1.5301380
H	1.8651830	-2.0380060	1.5345480		H	1.9025130	2.3446660	0.9311100

Table S4. Cartesian coordinates of the TL–TCNE ground-state (S_0) structure optimized at the ωB97XD/aug-cc-pVDZ/PCM(CH_2Cl_2) level of theory

Atom	X	Y	Z
C	-0.5908820	1.3083920	-0.0410770
C	-1.5899330	0.3927920	0.0564610
C	-2.2234660	-0.1443470	-1.1128340
C	-0.1300780	1.7802500	-1.3151120
C	0.0144330	1.8830880	1.1255890
C	-2.0842610	-0.0367280	1.3326450
N	-2.7517250	-0.5596050	-2.0548890
N	0.2351910	2.1833720	-2.3364210
N	-2.5017730	-0.3635860	2.3611370
N	0.4961290	2.3680920	2.0592790
C	2.0860150	-0.4983250	-0.0061520
C	1.5700690	-1.0022130	1.1953840

C	0.5580600	-1.9606670	1.1948830
C	0.0402470	-2.4331360	-0.0130500
C	0.5532960	-1.9477100	-1.2183460
C	1.5648880	-0.9899180	-1.2119640
H	1.9585090	-0.6293460	2.1436520
H	0.1631500	-2.3320730	2.1399830
H	-0.7568500	-3.1761420	-0.0154890
H	0.1547620	-2.3093170	-2.1657700
H	1.9514290	-0.6078450	-2.1574780
C	3.1880790	0.5292940	-0.0065800
H	3.1975370	1.1033830	0.9278810
H	4.1678420	0.0418870	-0.1085590
H	3.0800590	1.2269040	-0.8470490

Table S5. Cartesian coordinates of the FB–TCNE ground-state (S_0) structure optimized at the ωB97XD/aug-cc-pVDZ/PCM(CH_2Cl_2) level of theory

Atom	X	Y	Z
C	-0.8478430	1.1941700	0.2823040
C	-1.6098410	0.2486100	-0.3267890
C	-1.6758930	0.1537240	-1.7564330
C	-0.0715970	2.1283500	-0.4797900
C	-0.8148320	1.3148130	1.7112850
C	-2.4084290	-0.6679100	0.4344470
N	-1.7427690	0.0870330	-2.9096280
N	0.5463780	2.8895570	-1.0942950
N	-3.0641290	-1.3974540	1.0481620
N	-0.8001020	1.4206660	2.8634060
C	1.7321250	-0.7511260	1.2536810
C	0.8256700	-1.8107940	1.2121890
C	0.4085630	-2.3358310	-0.0122060
C	0.8991020	-1.7985340	-1.2052020
C	1.8043030	-0.7371430	-1.1850010
C	2.2025390	-0.2418680	0.0493440
H	0.4423870	-2.2237750	2.1443220
H	-0.2974900	-3.1643480	-0.0373920
H	0.5737360	-2.2046030	-2.1619600
H	2.1965680	-0.3006570	-2.1015440
H	2.0726970	-0.3259780	2.1959550
F	3.0859170	0.7824420	0.0822370

Table S6. Cartesian coordinates of the BZ–TCNE CT_1-state distorted face-to-face structure optimized at the ωB97XD/aug-cc-pVDZ/PCM level of theory

	in CH_2Cl_2				in CCl_4			
Atom	X	Y	Z		Atom	X	Y	Z
C	1.5072710	-0.5047450	0.2731810		C	1.5345530	-0.4818420	0.3268310
C	0.7914980	0.5161380	0.9812610		C	0.7988260	0.5254120	1.0326910
C	-0.2356320	0.1932610	1.8849920		C	-0.2518450	0.1864140	1.9046690
C	1.2821030	-1.8708790	0.5503390		C	1.3078730	-1.8528500	0.5782360
C	2.4782300	-0.1735450	-0.6992880		C	2.4661310	-0.1369450	-0.6788740
C	1.0641410	1.8884640	0.7704390		C	1.0138290	1.9030440	0.7922180
N	-1.1376270	-0.0796840	2.5753390		N	-1.1819260	-0.0916990	2.5544540
N	1.0463960	-2.9911680	0.7664130		N	1.0565970	-2.9763310	0.7582760
N	1.2642540	3.0167910	0.5646070		N	1.1459970	3.0349200	0.5522590
N	3.2484660	0.1135990	-1.5243810		N	3.1885500	0.1558370	-1.5447030
C	-1.6178080	-1.4441310	-0.9651230		C	-1.5242610	-1.4624500	-1.0262370
C	-0.8016280	-0.6573070	-1.7665830		C	-0.7299450	-0.6163990	-1.7901410
C	-0.9126450	0.7827940	-1.7395490		C	-0.9015940	0.8168340	-1.7242120
C	-1.8473530	1.4088780	-0.9208450		C	-1.8721610	1.3779540	-0.9039860
C	-2.6336590	0.6237400	-0.0984380		C	-2.6306530	0.5352510	-0.1104520
C	-2.5119000	-0.8154470	-0.1147690		C	-2.4481970	-0.8977600	-0.1659080
H	-1.5346380	-2.5266810	-0.9832190		H	-1.3842880	-2.5385380	-1.0654260
H	-0.0829350	-1.1104210	-2.4450770		H	0.0292030	-1.0175280	-2.4573180
H	-0.2597800	1.3674530	-2.3836510		H	-0.2543290	1.4476890	-2.3293710
H	-1.9336000	2.4915670	-0.9093110		H	-1.9975660	2.4557800	-0.8543610
H	-3.3629520	1.0748140	0.5698780		H	-3.3762910	0.9358640	0.5719950
H	-3.1522250	-1.3968260	0.5438380		H	-3.0565900	-1.5223300	0.4834790

Table S7. Cartesian coordinates of the BZ–TCNE CT_1-state T-shaped structure optimized at the ωB97XD/aug-cc-pVDZ/PCM level of theory

	in CH_2Cl_2				in CCl_4			
Atom	X	Y	Z		Atom	X	Y	Z
C	1.1132980	-0.0000120	-0.0009890		C	1.1394970	-0.0000630	0.0016040
C	2.5411840	0.0002510	-0.0000040		C	2.5667500	0.0007100	-0.0002880
C	3.2616040	1.2159170	0.0005760		C	3.2852360	1.2181970	-0.0010730
C	0.3787530	1.1986720	-0.0015920		C	0.3928560	1.1928920	0.0028330
C	0.3792410	-1.1989710	-0.0018250		C	0.3944280	-1.1940490	0.0030280
C	3.2620440	-1.2151520	0.0004570		C	3.2864630	-1.2160450	-0.0011390
N	3.8357850	2.2291930	0.0009760		N	3.8546280	2.2340130	-0.0016810
N	-0.2205430	2.2032000	-0.0021410		N	-0.2353760	2.1793170	0.0040410
N	3.8365870	-2.2282240	0.0015660		N	3.8569470	-2.2312470	-0.0020780
N	-0.2200200	-2.2035380	-0.0026010		N	-0.2321330	-2.1815250	0.0045070
C	-2.7688730	-0.0009200	-1.4281240		C	-2.7931950	0.0036270	-1.4302500

C	-2.7633530	-1.1931130	-0.7195500		C	-2.7829940	-1.1907070	-0.7262770
C	-2.7632650	-1.1923990	0.7225470		C	-2.7843170	-1.1947770	0.7159300
C	-2.7677110	0.0005240	1.4298110		C	-2.7952850	-0.0043100	1.4272390
C	-2.7647520	1.1927180	0.7212320		C	-2.7866300	1.1900660	0.7232820
C	-2.7654810	1.1919780	-0.7208540		C	-2.7859170	1.1941610	-0.7189430
H	-2.7741610	-0.0015080	-2.5143400		H	-2.7912470	0.0070650	-2.5166600
H	-2.7805580	-2.1479430	-1.2376300		H	-2.7822420	-2.1444960	-1.2465760
H	-2.7819240	-2.1465790	1.2418260		H	-2.7843680	-2.1515400	1.2306980
H	-2.7724940	0.0011180	2.5160290		H	-2.7943660	-0.0077530	2.5136480
H	-2.7842890	2.1474100	1.2395380		H	-2.7889860	2.1438040	1.2436420
H	-2.7853650	2.1461270	-1.2401370		H	-2.7885900	2.1508010	-1.2339570

Table S8. Cartesian coordinates of the TL–TCNE CT_1-state structure in CH_2Cl_2 optimized at the ωB97XD/aug-cc-pVDZ/PCM level of theory

Atom	X	Y	Z
C	0.7692190	-0.5001060	1.1677880
C	1.4567820	-0.8544810	-0.0385280
C	2.4887170	-0.0405630	-0.5539170
C	1.0716160	0.6952870	1.8581020
C	-0.2441030	-1.3240560	1.6958750
C	1.1261840	-2.0347960	-0.7395620
N	3.3068640	0.6708670	-0.9818040
N	1.2963690	1.7075220	2.3889770
N	0.8021250	-2.9934870	-1.3167150
N	-1.1197200	-1.9863760	2.0901840
C	-0.8120110	1.5416680	-0.8310710
C	-0.8932800	0.4496140	-1.7702510
C	-1.7718030	-0.5845480	-1.5660540
C	-2.5927980	-0.5692660	-0.4240770
C	-2.5668810	0.5275260	0.4891200
C	-1.6940340	1.5609790	0.2890500
H	-1.8221840	-1.4179850	-2.2607520
H	-3.2772550	-1.3938210	-0.2393660
H	-3.2344180	0.5179310	1.3467680
H	-1.6445090	2.3956610	0.9839210
H	-0.2399160	0.4639390	-2.6401520
C	0.1564640	2.6259960	-1.0680950
H	0.1696840	3.3652440	-0.2645830
H	1.1650420	2.2030310	-1.2135750
H	-0.0803450	3.1168050	-2.0270130

Table S9. Cartesian coordinates of the FB–TCNE CT_1-state face-to-face structure optimized at the ωB97XD/aug-cc-pVDZ/PCM level of theory

Atom	X	Y	Z
C	-0.7907220	0.5677260	-1.1419330
C	-1.5115400	0.6936170	0.0904750
C	-1.2779300	1.7853700	0.9548550
C	0.1910650	1.5055050	-1.5119460
C	-1.0236280	-0.5119270	-2.0253870
C	-2.5030410	-0.2449380	0.4573950
N	-1.0308520	2.6700520	1.6717020
N	1.0524070	2.2476130	-1.7774470
N	-3.2956080	-1.0415610	0.7629760
N	-1.1876930	-1.4255450	-2.7284970
C	2.4114520	-0.7563990	-0.6888360
C	2.6317460	0.4283990	0.0699230
C	1.9673560	0.6570240	1.3016210
C	1.0723030	-0.2671500	1.7691660
C	0.8422070	-1.4230680	0.9738440
C	1.5138910	-1.6891420	-0.2489590
H	2.9495300	-0.9011590	-1.6214650
H	3.3496400	1.1600480	-0.2903690
H	2.1673760	1.5664770	1.8606860
H	0.5316140	-0.1493370	2.7041780
H	1.2921700	-2.6045250	-0.7903940
F	-0.0218970	-2.2995030	1.4038800

References

1. Schmidt, B.; Laimgruber, S.; Zinth, W.; Gilch, P., A Broadband Kerr Shutter for Femtosecond Fluorescence Spectroscopy. *App. Phys. B* **2003,** *76*, 809-814.
2. Arzhantsev, S.; Maroncelli, M., Design and Characterization of a Femtosecond Fluorescence Spectrometer Based on Optical Kerr Gating. *Appl. Spectrosc.* **2005,** *59*, 206-220.
3. Nakamura, R.; Kanematsu, Y., Femtosecond Spectral Snapshots Based on Electronic Optical Kerr Effect. *Rev. Sci. Instrum.* **2004,** *75*, 636-644.
4. Chiu, C. C.; Hung, C. C.; Chen, C. L.; Cheng, P. Y., Ultrafast Time-Resolved Broadband Fluorescence Studies of the Benzene-Tetracyanoethylene Complex: Solvation, Vibrational Relaxation, and Charge Recombination Dynamics. *J. Phys. Chem. B* **2013,** *117*, 9734-9756.
5. Lorenc, M.; Ziolek, M.; Naskrecki, R.; Karolczak, J.; Kubicki, J.; Maciejewski, A., Artifacts in femtosecond transient absorption spectroscopy. *Appl. Phys. B* **2002,** *74*, 19-27.
6. Chiu, C. C.; Chen, W. C.; Cheng, P. Y., Excited-state vibrational relaxation and deactivation dynamics of trans-4-(N,N-dimethylamino)-4'-nitrostilbene in nonpolar solvents studied by ultrafast time-resolved broadband fluorescence spectroscopy. *J. Photochem. Photobiol., A* **2015,** *310*, 26-32.
7. Chiu, C.-C.; Hung, C.-C.; Cheng, P.-Y., Ultrafast Charge Recombination Dynamics in Ternary Electron Donor–Acceptor Complexes: (Benzene)2-Tetracyanoethylene Complexes. *J. Phys. Chem. B* **2016,** *120*, 12390-12403.
8. Singh, C.; Ghosh, R.; Mondal, J. A.; Palit, D. K., Excited state dynamics of a push-pull stilbene: A femtosecond transient absorption spectroscopic study. *J. Photochem. Photobiol., A* **2013,** *263*, 50-60.
9. Burns, L. A.; Vazquez-Mayagoitia, A.; Sumpter, B. G.; Sherrill, C. D., Density-Functional Approaches to Noncovalent Interactions: A Comparison of Dispersion Corrections (DFT-D), Exchange-Hole Dipole Moment (XDM) Theory, and Specialized Functionals. *J. Chem. Phys.* **2011,** *134*, 084107.
10. Li, A.; Muddana, H. S.; Gilson, M. K., Quantum Mechanical Calculation of Noncovalent Interactions: a Large-Scale Evaluation of PMx, DFT, and SAPT Approaches. *J. Chem. Theory Comput.* **2014,** *10*, 1563-1575.
11. Chai, J. D.; Head-Gordon, M., Long-Range Corrected Hybrid Density Functionals with Damped Atom-Atom Dispersion Corrections. *Phys. Chem. Chem. Phys.* **2008,** *10*, 6615-6620.
12. Tomasi, J.; Mennucci, B.; Cammi, R., Quantum mechanical continuum solvation models. *Chem. Rev.* **2005,** *105*, 2999-3093.
13. Frisch, M. J.; Trucks, G. W.; Schlegel, H. B.; Scuseria, G. E.; Robb, M. A.; Cheeseman, J. R.; Scalmani, G.; Barone, V.; Petersson, G. A.; Nakatsuji, H.*, et al.*, *Gaussian 16, Revision B.01*. Gaussian, Inc.: Wallingford CT, 2016.
14. Rumble, C. A.; Vauthey, E., Structural dynamics of an excited donor–acceptor complex

from ultrafast polarized infrared spectroscopy, molecular dynamics simulations, and quantum chemical calculations. *Phys. Chem. Chem. Phys.* **2019,** *21*, 11797-11809.
