# Supplementary material for: Concurrent Generation of Tight and Loose Ion Pairs upon Charge-Transfer Excitation of Electron Donor–Acceptor Complexes in Solution
Source: J Phys Chem Lett. 2026 Jan 26;17(5):1436–46. doi: 10.1021/acs.jpclett.5c03709 (PMC12884513; doi:10.1021/acs.jpclett.5c03709)
Supplement: Supplementary file 2 [file jz5c03709_si_002.pdf]

jz-2025-037099.R1

Name: Peer Review Information for "Concurrent Generation of Tight and Loose Ion Pairs upon Charge-Transfer Excitation of Electron Donor-Acceptor Complexes in Solution"

First Round of Reviewer Comments

Reviewer: 1

Comments to the Author

This manuscript reports on an investigation of the excited state dynamics of several electron donor-acceptor complexes using a combination of transient electronic absorption and emission spectroscopies. The authors observe a systematically shorter excited-state lifetime using fluorescence than transient absorption. This is explained in term of a distribution of ground-state DA pairs with different electronic coupling. Upon excitation, the most coupled pairs are fluorescent and short lived, whereas the less coupled pair are essentially non-fluorescent and longer lived.

This is an interesting and well-performed study with high quality data and adequate analysis.

It confirmed the most recent investigations, which point to presence of distributions of ion pairs upon photoexcitation and not well-defined pairs are often proposed.

Given the current activity in photoinduced bimolecular electron transfer processes, this investigation should be of interest to a broad readership. Therefore, publication in JPCL is recommended once the following point addressed:

Figure 5b does not give the right impression that a broad distribution of D-A pairs are photoexcited. Maybe adding multiple vertical arrows of different thickness symbolising a variety of oscillator strengths would be better.

- The measurements were only done in DCM which is medium polar. The authors could discuss how this distribution of ion pairs would be affected by going in less or more polar solvents.

Reviewer: 2

Comments to the Author

“Concurrent Generation of Tight and Loose Ion Pairs upon Charge-Transfer Excitation of Electron Donor–Acceptor Complexes in Solution”

This manuscript presents a comprehensive and carefully executed ultrafast spectroscopic study combining time-resolved fluorescence (TRFL) and transient absorption (TA) to investigate charge-transfer (CT) dynamics in several donor–acceptor complexes involving TCNE. The central observation—systematically shorter CT lifetimes obtained from TRFL compared to TA—and the resulting proposal of a concurrent generation mechanism of tight and loose ion pairs (TIPs and LIPs) represents an important conceptual advance over the conventional sequential TIP→LIP model. The work is timely, addresses a long-standing inconsistency in the interpretation of ultrafast measurements of CT complexes, and is supported by extensive experimental data across multiple systems, solvents, and aggregation states (binary vs ternary complexes). The discussion is generally thorough and well grounded in prior literature. Overall, the manuscript is suitable for publication in JPCL after following revisions.

1. The proposed concurrent generation of TIPs and LIPs is plausible and well motivated by the persistent discrepancy between τF and τA. However, the argument remains largely circumstantial, based on the failure of the conventional sequential model. While this is acceptable for a Letters format, the authors are encouraged to:

- More explicitly state which experimental observables cannot be reconciled even with modified sequential mechanisms (e.g., solvent- or donor-dependent TIP→LIP rates).

- Clarify whether any kinetic modeling (even simplified) was attempted to quantitatively rule out sequential schemes.

2. The importance of large reorganization energies and local heating is convincingly argued. However: The timescale for vibrational cooling (~10 ps) is critical to the trapping of LIPs. Please clarify whether this timescale is independently extracted from the data or adopted from prior studies. A brief discussion on whether changing solvent viscosity (not

just polarity) would be expected to modulate the TIP/LIP branching ratio could strengthen the mechanistic insight.

3. The authors can be used the references related to the donor-acceptor charge transfer systems and effects on TPA properties ; J. Phys. Chem. B 2021, 125, 32, 9244–9259; J. Phys. Chem. B 2021, 125, 16, 4187–4203; J. Phys. Chem. C 2019, 123, 30, 18270–18282; Org. Chem. Front., 2025,12, 3344-3362; https://doi.org/10.1002/cplu.202500354; https://doi.org/10.1002/chem.202100611

4. Figures 4 and 5 are central to the manuscript. Consider briefly restating their key messages in the main text immediately before or after they are introduced.

5. While details are provided in the SI, a short statement in the main text justifying the number of components used in TA global fits (especially the fixed τ4A) would improve transparency.

6. The TDDFT/PCM results are useful for structural insight but should be described more cautiously. Please emphasize that the calculations are illustrative rather than definitive, especially given the discrepancy with MD predictions.

Reviewer: 3

Comments to the Author

The manuscript “Concurrent Generation of Tight and Loose Ion Pairs upon Charge-Transfer Excitation of Electron Donor-Acceptor Complexes in Solution.” Submitted by Cheng, et al. is not appropriate for publication in JPC Letters. The authors' describe transient absorption and emission experiments aimed at understanding the rapid relaxation process of excited CT complexes and the dynamics of ion-pair formation in their different forms. There have been several descriptions of TIP, CIP, LIP and SSIP over the years and this manuscript attempts to describe the interconversions of these ion-pairs in detail. The report of different transient behavior being observed in absorption and emission experiments is not new and its origin has been identified based on differences in transient species concentrations (emissive TIP and ‘dark” LIP for example) as the authors suggest. However, the transient descriptions of these species hinge on understanding the origins of the observed emission contributions. The authors’ report no less than four transient

components are needed to fit the emission data with five components required to fit the absorption data for BZ-TCNE in dichloromethane. Tau 3F for this complex 3.1 ps which is not reported in the TL and FB fits. Would it not be reasonable to assume the 4.5 and 5.3 ps decays belong to Tau 3F and Tau F is not observed? Support for this suggestion comes directly from the authors' figure 2.c and 2.g where a long emissive component is not fit for the 750 nm decays. Given the CT absorption spectra (S1) it is clear the emission should occur and long wavelengths – exactly where the data is not fit correctly. I also question the origin of the blue emission. CT emission spectra free from background emissions are notoriously difficult to obtain due to their low emission yields. Yet the FC state reported with very fast decays appears to be very large, particularly when compared to the ca 750-800 nm emissions. Such rapid and intense emission requires large oscillator strengths, which is not observed for CT transitions.

I fear the authors have miss-interpreted their decay traces and major additional effort will be required to decipher the true decay components.

Reviewer: 4

Comments to the Author

Dr. Po-Yuan Cheng and co-authors submitted a manuscript entitled "Concurrent Generation of Tight and Loose Ion Pairs upon Charge-Transfer Excitation of Electron Donor-Acceptor Complexes in Solution." In the manuscript, the authors explain the nature of the excited-state lifetimes of donor-acceptor compounds derived from time-resolved fluorescence and visible transient absorption measurements.

In this study, experimental methods for investigating excited-state dynamics were employed. For a qualitative interpretation of the observed features, the TDDFT approximation was applied.

This research is a continuation of the authors' previous work published in J. Phys. Chem. B (2013), J. Phys. Chem. B (2016), J. Photochem. Photobiol. A (2015) etc.

The first issue I noticed is the absence of a clear separation of the manuscript into standard sections such as Introduction, Discussion, and Conclusions. In addition, the large number of abbreviations makes the manuscript difficult to read and to follow the main narrative of the research.

The theoretical simulations were used only at a qualitative level. I would recommend going deeper in the use of theoretical approaches, particularly for evaluating fluorescence rates of the different compounds. This could be achieved using excited-state dynamics modules available in Gaussian or ORCA software. Non-adiabatic simulations are especially powerful tools for studying excited-state dynamics. I strongly recommend that the authors perform quantitative comparisons between theoretical predictions and experimental observations.

Author's Response to Peer Review Comments:

The Journal of Physical Chemistry Letters
Manuscript ID: jz-2025-037099

Author's Response to Peer Review Comments

I would like to express my sincere gratitude to all reviewers for their thorough evaluation of our manuscript and for their insightful comments, which have enabled us to improve our work. The following are our point-by-point responses. Comments from the reviewers are shown in black, and the author's responses are shown in blue. Statements describing changes in the manuscript are underlined, and the corresponding revised text in the manuscript is highlighted in red.

Reviewer: 1

Recommendation: This paper is publishable subject to minor revisions noted. Further review is not needed.
Comments:
This manuscript reports on an investigation of the excited state dynamics of several electron donor-acceptor complexes using a combination of transient electronic absorption and emission spectroscopies. The authors observe a systematically shorter excited-state lifetime using fluorescence than transient absorption. This is explained in term of a distribution of ground-state DA pairs with different electronic coupling. Upon excitation, the most coupled pairs are fluorescent and short lived, whereas the less coupled pair are essentially non-fluorescent and longer lived.

This is an interesting and well-performed study with high quality data and adequate analysis. It confirmed the most recent investigations, which point to presence of distributions of ion pairs upon photoexcitation and not well-defined pairs are often proposed.

Given the current activity in photoinduced bimolecular electron transfer processes, this investigation should be of interest to a broad readership. Therefore, publication in JPCL is recommended once the following point addressed:

>Figure 5b does not give the right impression that a broad distribution of D-A pairs are photoexcited. Maybe adding multiple vertical arrows of different thickness symbolising a variety of oscillator strengths would be better.

Response: We sincerely thank the reviewer for the positive comments and recommendation. Figure 5b has been revised accordingly: multiple vertical arrows of different thickness have been added to represent excitation of a broad distribution of D–A configurations with varying oscillator strengths, as suggested.

>The measurements were only done in DCM which is medium polar. The authors could discuss how this distribution of ion pairs would be affected by going in less or more polar solvents.

Response: We thank the reviewer for this constructive suggestion. We would like to point out that our measurements were performed not only in CH_2Cl_2 (DCM) but also in CCl_4, a nonpolar solvent (Figure 3). As described in the original manuscript, τ_F is also substantially shorter than τ_A in this nonpolar medium, and we have noted that this observation implies that the LIPs observed here are unlikely to be SSIPs, since they are not expected to be stabilized in nonpolar solvents.

We have not yet carried out measurements in more polar solvents, such as ACN, but we agree that this is an important direction. In highly polar solvents, the CR rates in both TIPs and LIPs are expected to accelerate due to reduced exothermicities. If CR remains slower than the initial parallel relaxation into TIPs and LIPs, a similar disparity between τ_F and τ_A should persist. However, if CR becomes faster than, or comparable to, the initial relaxation, branching into LIPs would be reduced and τ_F and τ_A may converge. To address this point, we have added a short paragraph before the concluding paragraph to discuss the expected behaviors in solvents of higher polarity and viscosity. The added text (second-to-last paragraph, page 13 in the revised manuscript) reads:

Finally, we note that the present work is limited to medium-polar and nonpolar solvents of low viscosity. In highly polar media, CR may become competitive with or even faster than the initial parallel relaxation, reducing the branching into LIPs. Likewise, in much more viscous solvents, a more rigid solvent cage would restrict sampling of configuration space and could also suppress LIP formation.

+++

Reviewer: 2

Recommendation: This paper may be publishable, but major revision is needed; I would like to be invited to review any future revision.

Comments:

This manuscript presents a comprehensive and carefully executed ultrafast spectroscopic study combining time-resolved fluorescence (TRFL) and transient absorption (TA) to investigate charge-transfer (CT) dynamics in several donor–acceptor complexes involving TCNE. The central observation—systematically shorter CT lifetimes obtained from TRFL compared to TA—and the resulting proposal of a concurrent generation mechanism of tight and loose ion pairs (TIPs and LIPs) represents an important conceptual advance over the conventional sequential TIP→LIP model. The work is timely, addresses a long-standing inconsistency in the interpretation of ultrafast measurements of CT complexes, and is supported by extensive experimental data across multiple systems, solvents, and aggregation states (binary vs ternary complexes). The discussion is generally thorough and well grounded in prior literature. Overall, the manuscript is suitable for publication in JPCL after following revisions.

Response: We sincerely thank the reviewer for the thorough evaluation and positive comments on our work.

1. The proposed concurrent generation of TIPs and LIPs is plausible and well motivated by the persistent discrepancy between τ_F and τ_A. However, the argument remains largely circumstantial, based on the failure of the conventional sequential model. While this is acceptable for a Letters format, the authors are encouraged to:

- More explicitly state which experimental observables cannot be reconciled even with modified sequential mechanisms (e.g., solvent- or donor-dependent TIP→LIP rates).

Response: I assume that the reviewer is referring to a "modified sequential mechanism" with a broad range of TIP→LIP rates that depend on solvent and/or donor. If so, our response is that we have explicitly stated in the original manuscript (2nd paragraph, page 8): "*regardless of the specific nature of the nonfluorescent LIPs, the conventional sequential model cannot simultaneously account for the different* τ_F *and* τ_A *observed for the three similar DAC systems in* CH_2Cl_2*.*" In the same paragraph, we provided a detailed argument based on the reasonable assumption that the three closely related IPs in the same solvent (CH_2Cl_2) should exhibit very similar TIP→LIP conversion rates. These points are also emphasized in the Abstract, where we stated: "----- *the*

CT-state lifetimes obtained from TRFL are consistently shorter than those from TA by factors of ~2–5. This disparity, together with fluorescence lifetimes of ~5–30 ps in dichloromethane, cannot be reconciled with the conventional assumption that CT excitation initially yields only emissive tight ion pairs (TIPs)." As such, we believe that the reviewer's concern has been adequately addressed in the original manuscript.

• Clarify whether any kinetic modeling (even simplified) was attempted to quantitatively rule out sequential schemes.

Response: It is difficult to distinguish the two mechanisms solely by kinetic modeling, especially given that the initial IP ensemble is not a single kinetic species. Instead, our argument is based on the experimentally extracted τ_F and τ_A values from global fits of data across several closely related systems, as discussed on page 8 and 9 of the manuscript. As an illustrative limiting case, one can consider a highly simplified sequential model in which TIP→
LIP interconversion is much slower than the fluorescence lifetimes (which is likely relevant for TL–TCNE and FB–TCNE in CH_2Cl_2). In this limit, TIP→LIP become negligible on the time scale of τ_F and can be neglected, and the mere observation of different τ_F and τ_A values already naturally and "quantitatively" rules out the sequential model. However, I prefer not to overemphasize such a simplification, as it may appear as a lack of generality.

2. The importance of large reorganization energies and local heating is convincingly argued. However: The timescale for vibrational cooling (~10 ps) is critical to the trapping of LIPs. Please clarify whether this timescale is independently extracted from the data or adopted from prior studies. A brief discussion on whether changing solvent viscosity (not just polarity) would be expected to modulate the TIP/LIP branching ratio could strengthen the mechanistic insight.

Response: The vibrational cooling time scale (~10 ps) for CT states studied here is derived from our TRFL spectra of BZ-TCNE in CH_2Cl_2 (Figures 1a and 1c), which were analyzed in our previous work (JPCB 2013, 117, 9734-9756) by evaluating the time-dependent mean emission frequency. Similar vibrational relaxation/cooling time scales have also been reported by Rumble and Vauthey using vis-TA and TRIR spectroscopies (*PCCP* 2019, 21, 11797-11809). These references have been cited in the revised manuscript to indicate the origin of the ~10 ps timescale for vibrational cooling.

In response to the reviewer's suggestion regarding solvent viscosity, we have added a short paragraph before the concluding paragraph to discuss expected behaviors in solvents of higher viscosity. The added text (second-to-last paragraph, page 13 in the revised manuscript) reads: Finally, we note that the present work is limited to medium-polar and nonpolar solvents of low

viscosity. In highly polar media, CR may become competitive with or even faster than the initial parallel relaxation, reducing the branching into LIPs. Likewise, in much more viscous solvents, a more rigid solvent cage would restrict sampling of configuration space and could also suppress LIP formation.

3. The authors can be used the references related to the donor-acceptor charge transfer systems and effects on TPA properties; J. Phys. Chem. B 2021, 125, 32, 9244–9259; J. Phys. Chem. B 2021, 125, 16, 4187–4203; J. Phys. Chem. C 2019, 123, 30, 18270–18282; Org. Chem. Front., 2025,12, 3344-3362; https://doi.org/10.1002/cplu.202500354; https://doi.org/10.1002/chem.202100611

Response: Three of the references suggested by the reviewer have been added in the revised manuscript (refs. 5, 6, 7 in revised version).

4. Figures 4 and 5 are central to the manuscript. Consider briefly restating their key messages in the main text immediately before or after they are introduced.

Response: We appreciate the reviewer's emphasis on the importance of Figures 4 and 5. In the current version, their key messages are already summarized in the surrounding text and in the figure captions. Given the strict length limit of JPCLett, we have opted not to add further restatement in order to keep the manuscript within the allowed word count while preserving essential technical content.

5. While details are provided in the SI, a short statement in the main text justifying the number of components used in TA global fits (especially the fixed τ4A) would improve transparency.

Response: In the revised manuscript, the sentence where global fitting is first mentioned has been modified to state that the number of exponential components is guided by singular-value decomposition analysis (page 3). The added text now reads:

", and singular value decomposition indicated that at least four exponential components are required to describe the data."

6. The TDDFT/PCM results are useful for structural insight but should be described more cautiously. Please emphasize that the calculations are illustrative rather than definitive, especially given the discrepancy with MD predictions.

Response: In the original manuscript, we already noted several limitations of the TDDFT/PCM approach for ion pairs, including its likely underestimation of solvation stabilization due to the

absence of specific solvation effects and dynamical friction of the solvent cage (first paragraph on page 11 and first paragraph on page 12). We also pointed out that TDDFT/PCM and MD simulations may be viewed as complementary, and that "each method favors one structure over the other" (first paragraph on page 11).

However, to address the reviewer's concern, we have added a sentence in the revised manuscript to more explicitly state that the TDDFT/PCM results are intended to be illustrative rather than definitive. The added text (page 10) reads:

It should be emphasized that the TDDFT/PCM results are intended to provide qualitative structural insight rather than definitive structural assignments.

++

Reviewer: 3

Recommendation: This paper is not recommended because it does not provide new physical insights.

Comments:

>The manuscript "Concurrent Generation of Tight and Loose Ion Pairs upon Charge-Transfer Excitation of Electron Donor-Acceptor Complexes in Solution." Submitted by Cheng, et al. is not appropriate for publication in JPC Letters. The authors' describe transient absorption and emission experiments aimed at understanding the rapid relaxation process of excited CT complexes and the dynamics of ion-pair formation in their different forms. There have been several descriptions of TIP, CIP, LIP and SSIP over the years and this manuscript attempts to describe the interconversions of these ion-pairs in detail. The report of different transient behavior being observed in absorption and emission experiments is not new and its origin has been identified based on differences in transient species concentrations (emissive TIP and 'dark" LIP for example) as the authors suggest.

Response: We thank the reviewer for the critical comment and for highlighting the prior work on TIP, CIP, LIP, and SSIP. We fully agree that the existence and qualitative identification of emissive TIPs and "dark" LIP (SSIP) is not new, and we have cited and discussed these earlier studies. However, the conventional view has generally assumed that, upon CT excitation of DACs, emissive TIPs are formed first and then undergo slower, sequential conversion to LIPs of various forms.

The main contribution of this work is to show, based on a systematic comparison of τ_F and τ_A across several closely related DAC systems, that the purely sequential picture cannot account for the observed disparity. Our data suggest that TIPs and LIPs must be generated concurrently from a locally hot, structurally diverse initial ion-pair ensemble. Thus, while the TIP/LIP

terminology is established, the demonstration that concurrent TIP/LIP formation is a robust and general feature of CT excitation of DACs is, in our view, the central new aspect of this study.

>However, the transient descriptions of these species hinge on understanding the origins of the observed emission contributions. The authors' report no less than four transient components are needed to fit the emission data with five components required to fit the absorption data for BZ-TCNE in dichloromethane. Tau 3F for this complex 3.1 ps which is not reported in the TL and FB fits. Would it not be reasonable to assume the 4.5 and 5.3 ps decays belong to Tau 3F and Tau F is not observed? Support for this suggestion comes directly from the authors' figure 2.c and 2.g where a long emissive component is not fit for the 750 nm decays. Given the CT absorption spectra (S1) it is clear the emission should occur and long wavelengths – exactly where the data is not fit correctly.

Response: First, we would like to emphasize that our central conclusion does not hinge solely on understanding the origins of the observed emission, but more fundamentally on the systematic difference between the lifetimes obtained from time-resolved emission and absorption.

Regarding the specific point about TL–TCNE and FB–TCNE, the reviewer indeed raised an important question. The suggestion is that the ~5 ps decay in these systems might correspond to τ_{3F} (vibrational/structural relaxation), as in BZ–TCNE, and that the genuine τ_F of the relaxed CT state is not observed simply because its emission is too weak. However, this scenario implies that vibrational/structural relaxation brings the system into a state whose emission is essentially undetectable, i.e., a nonfluorescent IP. In that case, the relaxed nonfluorescent IP is effectively the LIP in the sequential model and should be observed in TA, and the "genuine τ_F" would coincide with τ_A. This is exactly the situation we discuss on page 8, where we stated: "*Conversely, if the short τ_F (~5 ps) of TL–TCNE and FB–TCNE TIPs were dictated by rapid TIP→LIP conversion, then an equally rapid conversion should also occur in BZ–TCNE and produce a similarly short τ_F, which again contradicts our observations.*"

In other word, if any intracomplex vibrational/structural relaxation were so efficient to quench essentially all fluorescent IP population into a nonfluorescent state within ~5 ps in TL–TCNE and FB–TCNE, the same process should also rapidly occur in BZ-TCNE in the same solvent and quench its fluorescence, which we did not observe. Thus, the above reasoning rules out such scenario and indicates that it is the much faster CR in TL-TCNE and FB-TCNE that is responsible for quenching the emissive CT-state population, rather than an unusual intracomplex relaxation process. A minor support to these arguments is that the calculated oscillator strengths of the relaxed CT_1 states of BZ–TCNE, TL–TCNE, and FB–TCNE are of the same order of

magnitude (see Table2). If the emissive CT states of TL–TCNE and FB– TCNE could survive to full relaxation, their fluorescence should be detectable.

The reviewer also suggests that a longer emissive component at 750 nm (or 690 nm) that is not well reproduced in the global fits might correspond to an unreported longer τ_F. However, these long-wavelength emission components contribute only negligibly to the total fluorescence. The fluorescence traces shown in Figure 2 are normalized, which visually exaggerates the relative weight of the long-wavelength emission. Consequently, these minor long-wavelength contributions are too small to be represented in the global fits.

More importantly, even these weak long-wavelength emission transients decay significantly faster than the corresponding TA signal. For TL–TCNE (Figures 2c and 2d), at ~20 ps the 750 nm emission has decayed to a negligible level, whereas the TA signal at 450 nm still retains roughly one third of its maximum amplitude. Similarly, for FB–TCNE (Figures 2g and 2h), at ~30 ps the 690 nm emission has essentially vanished, while the 450 nm TA signal remains at about half of its maximum.

>I also question the origin of the blue emission. CT emission spectra free from background emissions are notoriously difficult to obtain due to their low emission yields. Yet the FC state reported with very fast decays appears to be very large, particularly when compared to the ca 750-800 nm emissions. Such rapid and intense emission requires large oscillator strengths, which is not observed for CT transitions. I fear the authors have miss-interpreted their decay traces and major additional effort will be required to decipher the true decay components.

Response: I assume that the reviewer's concern about the difficulty of obtaining CT emission spectra free from background refers primarily to the steady-state emission measurements. Because of their low emission yields, steady-state CT emission spectra of DACs are indeed prone to contamination from trace impurities with long fluorescence lifetimes. This issue is common to all low-emission-yield systems, not just DACs.

However, in ultrafast TRFL with a gating window of ~0.2 ps, this problem is greatly reduced: long-lived impurity fluorescence contributes negligibly within such an ultrashort time window, even if its steady-state intensity is comparable or larger than that of the CT emission. Thus, for systems with very low emission yields due to ultrafast lifetimes, the gated emission signal from the CT state can still be quite strong, while the contribution from long-live impurity fluorescence remains negligibly. In our TRFL measurements, we always verified that there is no detectable background emission from donor-only or acceptor-only solutions over the time window studied. The only significant background in the early-time TRFL spectra is solvent Raman scattering, which partially overlaps with the earliest emission. This contribution is systematically removed

using the background subtraction procedure described in our previous work (JPCB 2013, 117, 9734–9756).

The reviewer also commented that "the FC state reported with very fast decays appears to be very large, particularly when compared to the ca 750-800 nm emissions. Such rapid and intense emission requires large oscillator strengths, which is not observed for CT transitions." This behavior is, in fact, physically reasonable. The initial CT emission from the FC region reflects the same relatively large oscillator strength as the CT absorption (see Table 2). This initially "bright" FC state undergoes ultrafast solvation and vibrational relaxation into the relaxed CT state (TIP), whose emission is red-shifted and has a smaller oscillator strength (see Table 2). Consequently, the blue emission decays very rapidly (quenched by solvation and structural relaxation), whereas the weaker red emission persists longer in time. In CH_2Cl_2, the intense blue emission exists for less than ~1 ps, while the weak red emission extends over a much longer timescale, so the steady-state fluorescence spectrum actually does not exhibit an anomalously strong blue band (see our previous work JPCB 2013, 117, 9734–9756).

These temporal features of CT emission from BZ–TCNE have been analyzed and discussed in detail in our earlier work (JPCB 2013, 117, 9734–9756). To clarify this picture visually, we have slightly revised Figure 4 in the manuscript, using arrows of varying length and color (blue to red) to schematically represent the evolution of emission strength and wavelength from the initial IP/FC ensemble to the relaxed TIP.

+++

Reviewer: 4

Recommendation: This paper may be publishable, but major revision is needed; I would like to be invited to review any future revision.

Comments:

Dr. Po-Yuan Cheng and co-authors submitted a manuscript entitled "Concurrent Generation of Tight and Loose Ion Pairs upon Charge-Transfer Excitation of Electron Donor-Acceptor Complexes in Solution." In the manuscript, the authors explain the nature of the excited-state lifetimes of donor-acceptor compounds derived from time-resolved fluorescence and visible transient absorption measurements.

In this study, experimental methods for investigating excited-state dynamics were employed.

For a qualitative interpretation of the observed features, the TDDFT approximation was applied.

This research is a continuation of the authors' previous work published in J. Phys. Chem. B (2013), J. Phys. Chem. B (2016), J. Photochem. Photobiol. A (2015) etc.

The first issue I noticed is the absence of a clear separation of the manuscript into standard sections such as Introduction, Discussion, and Conclusions. In addition, the large number of abbreviations makes the manuscript difficult to read and to follow the main narrative of the research.

Response: We thank the reviewer for these comments. The reason that the manuscript is not separated into standard sections (Introduction, Discussion, and Conclusions) is simply because we have to comply with the format requirement of JPCLett, which does not allow section headings in Letters! Within this constraint, we have structured the manuscript to follow a clear logical progression from introduction through results, discussion, and conclusion in continuous prose.

Concerning the use of abbreviations, terms such as TRFL, TA, DAC, CT, CR, IP, TIP, LIP, and MD are standard in this research field and are defined upon first use. Given the strict 4000-word limit for JPCL, employing these abbreviations is also helpful to keep the text concise; the original manuscript is already close to this limit (~3995 words, excluding figure captions). Nevertheless, we agree that nonstandard abbreviations should be minimized. In the revised version, we have removed the abbreviation "RV" for "Rumble and Vauthey" and now use their names explicitly.

The theoretical simulations were used only at a qualitative level. I would recommend going deeper in the use of theoretical approaches, particularly for evaluating fluorescence rates of the different compounds. This could be achieved using excited-state dynamics modules available in Gaussian or ORCA software. Non-adiabatic simulations are especially powerful tools for studying excited-state dynamics. I strongly recommend that the authors perform quantitative comparisons between theoretical predictions and experimental observations.

Response: The CT_1-S_0 oscillator strengths, which are proportional to the radiative decay rates, for both FC and relaxed CT states of BZ–TCNE, TL–TCNE, and FB–TCNE have already been calculated and are given in Table 2. We agree that more extensive theoretical work, including nonadiabatic excited-state dynamics, would be highly informative for a deeper understanding of the CR dynamics of TIPs and LIPs in these DAC systems.

However, while such simulations are important for a more detailed mechanistic picture, they are not essential for the central conclusion of this Letter, which is based on the robust experimental disparity between τ_F and τ_A. In addition, incorporating nonadiabatic dynamics results would substantially increase the length and complexity of the manuscript, which is already at the 4000-word limit for JPCL, and would risk diverting the focus from the main experimental message.

jz-2025-037099.R2

Name: Peer Review Information for "Concurrent Generation of Tight and Loose Ion Pairs upon Charge-Transfer Excitation of Electron Donor-Acceptor Complexes in Solution"

Second Round of Reviewer Comments

Reviewer: 2

Comments to the Author

The manuscript enhanced the reviewers suggestions. It can be acceppted for publication.

Reviewer: 4

Comments to the Author

The manuscript can be accepted in the current form.

Reviewer: 1

Comments to the Author

My comments have been convincingly addressed and, thus, I recommend publication of this ms in its present form.

Author's Response to Peer Review Comments:

All colored text markups have been removed in the uploaded revised manuscript.

jz-2025-037099.R3

Name: Peer Review Information for "Concurrent Generation of Tight and Loose Ion Pairs upon Charge-Transfer Excitation of Electron Donor-Acceptor Complexes in Solution"

Third Round of Reviewer Comments

Reviewer 3:

The revised manuscript “Concurrent Generation of Tight and Loose Ion Pairs upon Charge-Transfer Excitation of Electron Donor-Acceptor Complexes in Solution.” Submitted by Cheng, et al. has not considered my earlier comments and, as a result, is still not appropriate for publication in JPC Letters. The authors' describe transient absorption and emission experiments aimed at understanding the rapid relaxation process of excited CT complexes and the dynamics of ion-pair formation in their different forms.

I must admit the difficulty may be due to my poorly framed initial responses. I try not to delve into detail in my responses because this typically leads to hypothetical scenarios to demonstrate a specific point. Authors then tend to respond to the hypothetical scenario and ignore the underlying problem. In this case I will try to be blunt.

The transient descriptions of all IP species hinge on understanding the origins of the observed emission and absorption contributions. For the BZ–TCNE TRFL data the authors’ report decay components of 0.2, 0.6, and 3.1 ps as well as 29 ps. The TA data yields <0.2, 0.5, 4.1, 29 ps as well as 62 ps. Clearly there is agreement between the TRFL and TA data if one assumes species responsible for the 62 ps contribution to the TA is formed irreversibly. As an example only, consider the process defined by the dashed arrow in Figure 4 may be slow. However, I am reluctant to label any of these processes directly with the *rate constants* used in Figure 4 because the integrated rate expressions implied by both mechanisms will give complicated functions for each of the experimentally determined decay times. Moving forward to the TL–TCNE (and FB–TCNE) TRFL data we get decays of ,<0.2 (0.2), 0.7 (0.6), 4.5 (5.3) ps – very similar to the fastest 3 decays observed in the BZ-TCNE complex. The TA data yields decays of ,<0.2 (<0.2), 0.7 (NA), 4.2 (4.4), 10.5 (27) again very similar to the BZ-TCNE data if one assumes the longest TA contribution does not occur. Also note that I will discuss the absence of the 10.5 (27) ps decay in paragraph below. As

an example only, for the TL and BF-TCNE complexes the IPs may decay faster than the LIP is produced. While I give example solutions to the decay processes, it is not my responsibility to reassign the authors' decay processes although I could give multiple scenarios that account for these data. Most importantly, there are multiple scenarios in which these decays can be assigned, none of which require direct formation of the LIP. I state again, the transient descriptions of all IP species hinge on understanding origins of the observed emission and absorption contributions. The ca 5 ps decay components in the transient data need not indicate rapid TIP – LIP conversion and it does occur in both TA and TRFL decays for all 3 DACs studied and could be due to some other relaxation/conversion (of which I can suggest several). The authors' aim was to show that the sequential mechanism in Figure 4 was not sufficient to describe the observed decays and that concurrent formation of TIP and LIP were required to describe their data. Both of these statements are likely false I strongly urge rejection of this manuscript.

In the above discussion I suggested that the 29 decays could be due to decay of TIP via CR and production of LIP. For the remaining DACs, the 10.5 and 27 ps TA decays could be assigned similarly but were not recorded for the TRFL data. Observation of a long-lived species in TRFL for both DACs in the long-wavelength end of the data shown in Figure 3. The authors' response to my earlier suggestion concerning the importance of these decays was akin to "these are not the decays you are looking for". Clearly this statement is false. These decays must be considered. I urge the authors to use their CT absorption data to estimate the corresponding relaxed CIP emission spectra. This process has been described in many reports, perhaps most notably by Farid and his collaborators.

Author's Response to Peer Review Comments:

The Journal of Physical Chemistry Letters

Manuscript ID: jz-2025-037099

Author's 2nd Response to Reviewer 3's Comments

Note: Comments from the reviewer are shown in black, and the author's responses are shown in blue.

Reviewer: 3

Comments:

The revised manuscript "Concurrent Generation of Tight and Loose Ion Pairs upon Charge-Transfer Excitation of Electron Donor-Acceptor Complexes in Solution." Submitted by Cheng, et al. has not considered my earlier comments and, as a result, is still not appropriate for publication in JPC Letters. The authors' describe transient absorption and emission experiments aimed at understanding the rapid relaxation process of excited CT complexes and the dynamics of ion-pair formation in their different forms.

I must admit the difficulty may be due to my poorly framed initial responses. I try not to delve into detail in my responses because this typically leads to hypothetical scenarios to demonstrate a specific point. Authors then tend to respond to the hypothetical scenario and ignore the underlying problem. In this case I will try to be blunt.

The transient descriptions of all IP species hinge on understanding the origins of the observed emission and absorption contributions. For the BZ–TCNE TRFL data the authors' report decay components of 0.2, 0.6, and 3.1 ps as well as 29 ps. The TA data yields <0.2, 0.5, 4.1, 29 ps as well as 62 ps. Clearly there is agreement between the TRFL and TA data if one assumes species responsible for the 62 ps contribution to the TA is formed irreversibly. As an example only, consider the process defined by the dashed arrow in Figure 4 may be slow. However, I am reluctant to label any of these processes directly with the *rate constants* used in Figure 4 because the integrated rate expressions implied by both mechanisms will give complicated functions for each of the experimentally determined decay times. Moving forward to the TL–TCNE (and FB–TCNE) TRFL data we get decays of ,<0.2 (0.2), 0.7 (0.6), 4.5 (5.3) ps – very similar to the fastest 3 decays observed in the BZ-TCNE complex. The TA data yields decays of ,<0.2 (<0.2), 0.7 (NA), 4.2 (4.4), 10.5 (27) again very similar to the BZ-TCNE data if one assumes the longest TA contribution does not occur. Also note that I will discuss the absence of the 10.5 (27) ps decay in paragraph below. As an example only, for the TL and BF-TCNE complexes the IPs may decay faster than the LIP is produced. While I give example solutions to the decay processes, it is not my responsibility to reassign the authors' decay processes although I could give multiple scenarios that account for these data. Most importantly, there are multiple scenarios in which these decays can be assigned, none of which require direct formation of the LIP. I state again, the transient descriptions of all IP species hinge on understanding origins of the observed emission and absorption contributions. The ca 5 ps decay components in

the transient data need not indicate rapid TIP – LIP conversion and it does occur in both TA and TRFL decays for all 3 DACs studied and could be due to some other relaxation/conversion (of which I can suggest several). The authors' aim was to show that the sequential mechanism in Figure 4 was not sufficient to describe the observed decays and that concurrent formation of TIP and LIP were required to describe their data. Both of these statements are likely false I strongly urge rejection of this manuscript.

In the above discussion I suggested that the 29 decays could be due to decay of TIP via CR and production of LIP. For the remaining DACs, the 10.5 and 27 ps TA decays could be assigned similarly but were not recorded for the TRFL data. Observation of a long-lived species in TRFL for both DACs in the long-wavelength end of the data shown in Figure 3. The authors' response to my earlier suggestion concerning the importance of these decays was akin to "these are not the decays you are looking for". Clearly this statement is false. These decays must be considered. I urge the authors to use their CT absorption data to estimate the corresponding relaxed CIP emission spectra. This process has been described in many reports, perhaps most notably by Farid and his collaborators.

Response: We would like to thank Reviewer 3 for the critical comments. I would like to begin by reiterating the key point we aimed to emphasize, stated both in the main text as well as in the abstract: "This disparity, together with fluorescence lifetimes of ~5–30 ps in dichloromethane, cannot be reconciled with the conventional assumption that CT excitation initially yields only emissive tight ion pairs (TIPs)." In our view, Reviewer 3 tried to interpret our data on a case-by-case basis within the framework of the sequential mechanism, whereas our argument is primarily built on the collective behavior across all systems. It may indeed be possible to rationalize an individual system using the sequential mechanism by adjusting rate constants to reproduce the observed dynamics, but inconsistencies emerge once all cases are considered together. We would also like to point out that the "example solutions (assignments)" suggested by Reviewer 3, as discussed below, are essentially encompassed by the reasoning already presented in the manuscript (page 8, revised version), where we argue that the sequential mechanism is incompatible with our combined TRFL and TA observations.

As the reviewer noted for BZ–TCNE in CH_2Cl_2, one can obtain apparent agreement between TRFL and TA data "if one assumes species responsible for the 62 ps contribution to the TA is formed irreversibly. As an example only, consider the process defined by the dashed arrow in Figure 4 may be slow," and that "the 29 ps decay could be due to decay of TIP via CR and production of LIP." It is important to note that making such an assignment within the framework of the sequential mechanism implies that the TIP→LIP conversion must be slower, and possibly much slower, than ~30 ps in CH_2Cl_2. As an example only, a reasonable 50/50 branching between CR and TIP→LIP would imply time constants of ~60 ps for both processes.

The reviewer then moved forward to the TL–TCNE and FB–TCNE cases, stating that "As an example only, for the TL and FB-TCNE complexes the IPs may decay faster than the LIP is produced." I assume that this assignment is still within the sequential mechanism framework, as the reviewer insisted. The TL–TCNE and FB–TCNE IPs may indeed decay faster than LIP production, such that their fluorescence lifetimes (~5 ps) are dictated by CR. (Recall that interpreting the BZ–TCNE in CH_2Cl_2 data within the same sequential framework leads to the conclusion that TIP→LIP conversion must be slower, and possibly much slower, than ~30 ps). However, in such a scenario, LIP production would be minimal or even negligible if only the sequential mechanism were operative, and one would then expect TRFL and TA to report nearly identical lifetimes, contrary to our observations.

One might argue that even a small LIP branching could still account for the τ_A observed in TA measurements. However, the TA detection of IPs in this work relies primarily on the resonance absorption of the TCNE anion in both TIPs and LIPs, which are typically assumed to have similar oscillator strengths, and the DAS spectra shown in Figure S5 clearly indicate that the long-lived LIP-associated components are far from negligible compared to those assignable to TIPs.

To reconcile these observations for TL–TCNE and FB–TCNE within the sequential framework, one must assume a substantial branching into LIPs in competition with the fast-decaying TIPs. As an illustrative example, a 50/50 branching between CR and TIP→LIP would imply time constants of ~10 ps for both processes, and one would need to explain why such intracomplex rearrangement is rapid for TL–TCNE and FB–TCNE but much slower for BZ–TCNE in the same solvent (CH_2Cl_2), despite the three DACs are closely related.

Moreover, the observation of distinct τ_F and τ_A implies that TIP→LIP conversion must be effectively irreversible, and one would also need to explain why the reversed process is much slower than the forward one (noting that, in this case, the LIPs are certainly not SSIPs). In addition, any such individually tailored pictures must also account for the lifetime disparity observed in nonpolar media and in ternary DAC systems.

Thus, while each individual system might be "rationalized" separately, the full set of data for the three closely related DACs in CH_2Cl_2, together with other systems studied, cannot be reconciled simultaneously within a conventional sequential framework. By contrast, as detailed in the manuscript, the concurrent generation mechanism provides a more coherent and unified description of the data for all systems.

The reviewer further stated that "The ca. 5 ps decay components in the transient data need not indicate rapid TIP–LIP conversion and it does occur in both TA and TRFL decays for all 3 DACs studied and could be due to some other relaxation/conversion." We do not fully understand this comment, because we never claimed in the manuscript that the ~5 ps decay components arise from TIP–LIP conversion. What we did state in the manuscript is: "if the short τ_F (~5 ps) of TL–TCNE and FB–TCNE TIPs were dictated by rapid TIP→LIP conversion,

then an equally rapid conversion should also occur in BZ–TCNE and produce a similarly short τ_F, which again contradicts our observations.” In other words, this statement is presented as a *proof by contradiction* argument to show the incompatibility between our data and the sequential mechanism: because the logical consequence contradicts experimental observations, the initial assumption (that the ~5 ps decay components are due to TIP–LIP conversion) must be false. Within our concurrent generation mechanism, the ~5 ps fluorescence lifetimes observed for TL–TCNE and FB–TCNE can be simply assigned to rapid nonequilibrium CR of their TIPs.

In the last part of the comment, the reviewer reiterated a concern already raised in the first round, namely that the 10.5 and 27 ps decays observed in the TA data of TL–TCNE and FB-TCNE “must” also appear in the corresponding TRFL data, particularly at long emission wavelengths. We disagree with this assertion. In our first-round response, we have explained that these slower long-wavelength emissions are too weak to be represented in the global fits and, more importantly, that the single-wavelength fluorescence transients at these long-wavelengths still decay significantly faster than TA signals. We believe that these arguments explain why the weak long-wavelength emission does not undermine our conclusion that τ_F and τ_A differ significantly and adequately address the reviewer’s concern. We would have appreciated a more specific critique of which part of our arguments the reviewer found unconvincing, so that we could address it directly, rather than a blanket statement that our earlier responses are all false.
